# ER proteostasis failure in HYOU1 deficiency alters B cells, neutrophils, and interferon signalling

**DOI:** 10.70962/jhi.20250234

**Published:** 2026-09-07

**Authors:** Aida Idani, Sabrina Bibi-Triki, Tristan Stemmelen, Mirjana Radosavljevic, Pierre Grenot, Aurélie Hirschler, Alice Bernard, Michelle Rosenzwajg, Jianying Yang, Alice Eischen, Lydie Naegely, Cécile Macquin, Angélique Pichot, Perrine Spinnhirny, Antoine Hanauer, Nasrin Alipour-Olayei, Thomas Cherrier, Aline Kolmer, Nicodème Paul, Rory J. Olson, Eva Morava, Zhichao Miao, Christine Carapito, Parastoo Rostami, Nima Parvaneh, Mohammad Shahrooei, Anne Molitor, Raphael Carapito, Seiamak Bahram

**Affiliations:** 1 Molecular Immuno-Rheumatology Laboratory (IRM), INSERM UMR_S 1109, GENOMAX-CYTOMAX Platforms, Immunology and Hematology Research Center, Strasbourg Research Center in Biomedicine (CRBS), Strasbourg Federation of Translational Medicine (FMTS), School of Medicine, University of Strasbourg, Strasbourg, France; 2 Strasbourg Interdisciplinary Thematic Institute (ITI) for Precision Medicine, TRANSPLANTEX NG, University of Strasbourg, Strasbourg, France; 3 Clinical Immunology Laboratory, Nouvel Hôpital Civil, Hôpitaux Universitaires de Strasbourg, Strasbourg, France; 4 Laboratoire de Spectrométrie de Masse BioOrganique, CNRS, IPHC, UMR 7178, Université de Strasbourg, Strasbourg, France; 5 Biotherapy (CIC-BTi) and Inflammation-Immunopathology-Biotherapy Department (i2B), Assistance Publique-Hôpitaux de Paris, Pitié-Salpêtrière Hospital, Biotherapy (Centre d’Investigation Clinique Intégré en Biothérapies & Immunologie), Paris, France; 6 Institut National de la Santé et de la Recherche Médicale UMR_S 959, Immunology-Immunopathology-Immunotherapy (i3), Sorbonne Université, Paris, France; 7 Hematology Laboratory, Hôpital de Hautepierre, University Hospital of Strasbourg, Strasbourg, France; 8 Center for Individualized Medicine, Mayo Clinic, Rochester, MN, USA; 9 Department of Genetics and Genomic Sciences, Icahn School of Medicine at Mount Sinai, New York, NY, USA; 10 GMU-GIBH Joint School of Life Sciences, The Guangdong-Hong Kong-Macau Joint Laboratory for Cell Fate Regulation and Diseases, Guangzhou National Laboratory, Guangzhou Medical University, Guangzhou, China; 11 Shanghai Key Laboratory of Anesthesiology and Brain Functional Modulation, Clinical Research Center for Anesthesiology and Perioperative Medicine Translational Research Institute of Brain and Brain-Like Intelligence, Shanghai Fourth People’s Hospital, School of Medicine, Tongji University, Shanghai, China; 12 Division of Endocrinology and Metabolism, Department of Pediatrics, Children’s Medical Center, Tehran University of Medical Sciences, Tehran, Iran; 13 Division of Allergy and Clinical Immunology, Department of Pediatrics, Children’s Medical Center, Tehran University of Medical Sciences, Tehran, Iran; 14 Dr. Shahrooei Laboratory, Tehran, Iran; 15 Institut Universitaire de France (IUF), Paris, France

## Abstract

Hypoxia upregulated 1 (HYOU1) is a stress-inducible ER chaperone. We investigated 2 unrelated patients carrying biallelic *HYOU1* variants and presenting with primary immunodeficiency. Patient 1, homozygous for p.Pro444His, displayed failure to thrive, hypoglycemia, B cell lymphopenia, and neutropenia. Patient 2, compound heterozygous for p.Arg262Gln and p.Pro757_Glu758insAla, exhibited recurrent infections, enteropathy, and hypogammaglobulinemia. In Patient 1, while *HYOU1* transcription was preserved, the protein was severely reduced. Tunicamycin treatment of dermal fibroblasts showed a blunted unfolded protein response and defective induction of ER stress–responsive genes. Immunophenotyping showed near-absence of circulating B cells, and single-cell RNA sequencing of bone marrow identified an arrest at the pro-B cell stage. Neutrophils displayed hypogranulation and dysregulated IFN- and apoptosis-associated transcriptional signatures, unresponsive to G-CSF. HYOU1 deficiency hence results in ER stress–induced proteostasis failure that simultaneously impairs adaptive immunity through B cell developmental arrest and innate immunity through neutrophil dysfunction and IFN pathway imbalance. This work expands the spectrum of HYOU1 deficiency and further identifies ER proteostasis as a central determinant of immune homeostasis.

## Teaser

Multi-omics characterization of 2 patients with biallelic HYOU1 variants reveals that HYOU1 deficiency impairs the unfolded protein response, disrupts early B cell development, and alters neutrophil granulation as well as IFN signalling, further establishing ER proteostasis as a contributor to human immune homeostasis.

## Graphical Abstract

**Figure:**
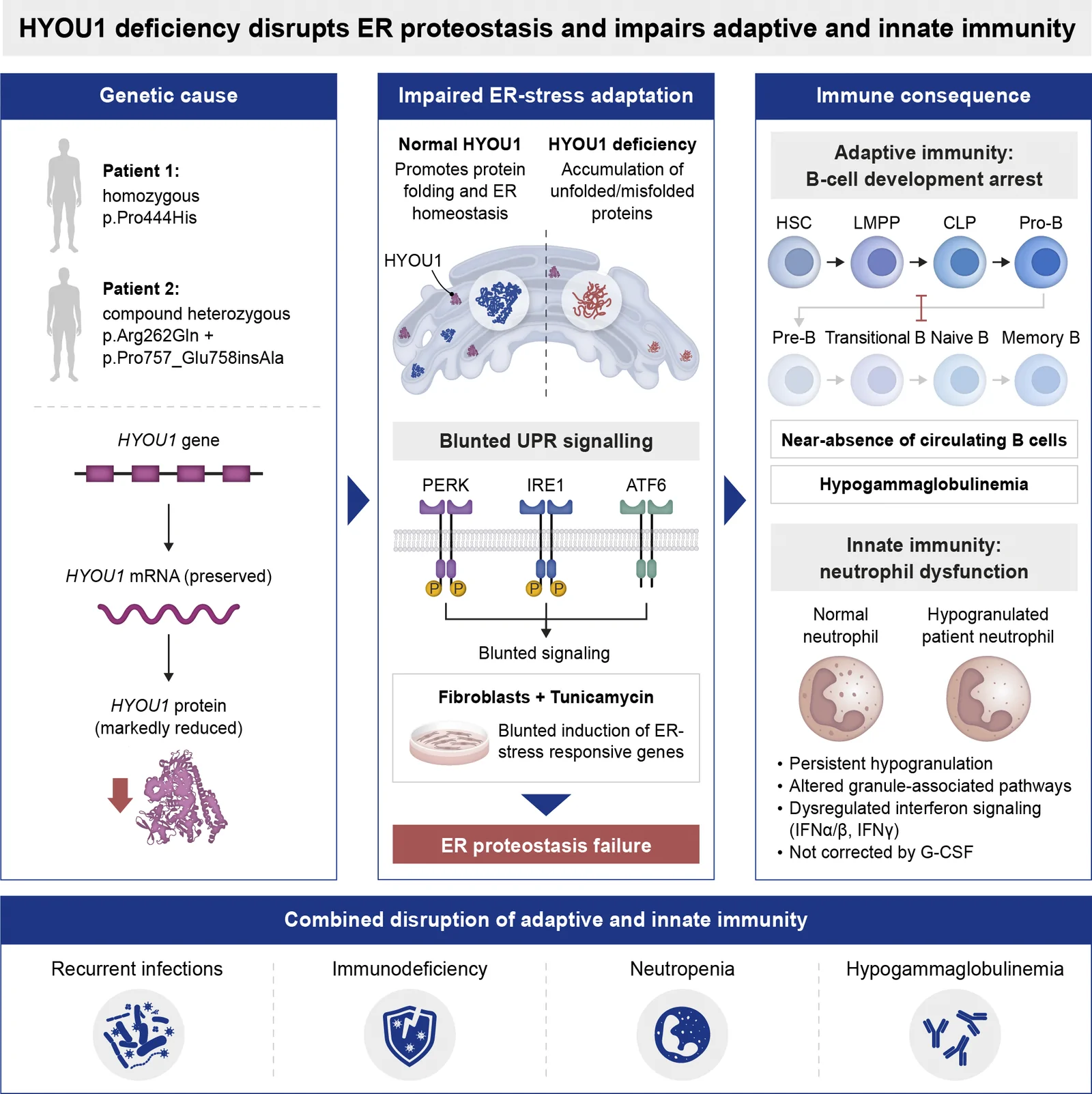

## Introduction

Hypoxia upregulated 1 (HYOU1), also known as oxygen-regulated protein 150 (ORP150) or glucose-regulated protein 170 (GRP170), belongs to the heat shock protein 70 (HSP70) family, involved in protecting cells in stress situations (1), including through the unfolded protein response (UPR) which is activated by the accumulation of unfolded and misfolded proteins in the ER (2). Once initiated, the UPR serves as a mechanism to manage ER stress and rescues the cell from apoptosis (3). Located on chromosome 11q23.3, the *HYOU1* gene encodes a 150-kDa protein, upregulated as a result of ER stress, hypoxia, and glucose deficiency, eventually playing a crucial role in managing ER stress and preventing apoptosis (4). At the organism level, HYOU1 is known to be involved in various ER stress–related diseases such as diabetes mellitus, neurodegenerative disorders, and cardiovascular diseases (5), yet its role in inborn errors of immunity (IEIs) remains largely unexplored. In 2017, Haapaniemi et al. reported the first patient (a 45-year-old woman) carrying compound heterozygous variants in *HYOU1*, associated with recurrent infections, stress-induced hypoglycemia, granulocytopenia, and B cell and dendritic cell loss, attributing the combined immune and metabolic phenotype to impaired UPR and mitochondrial function, and describing it as a novel immunometabolic disease (6). Their evaluations showed altered cellular metabolism, possibly resulting from impairments in the UPR and mitochondrial function. These findings were in line with the hitherto known functions of HYOU1 in protein folding and secretion in the ER (7). Both variants reported in the case were located within the HSP70 domain of HYOU1. Two subsequent reports identified 2 additional patients with recessive *HYOU1* variants presenting with pancytopenia, severe infections, and early lethality (8, 9). Based on these observations, HYOU1 deficiency is categorized among the 559 IEIs as a congenital defect of phagocyte number or function (OMIM #601746), as defined by the International Union of Immunological Societies Expert Committee (10).

Here, we describe two *HYOU1*-deficient patients with novel biallelic variants in the gene. Through a comprehensive multi-omics investigation, we delineate the molecular and cellular consequences of *HYOU1* deficiency, demonstrating its essential role in hematopoiesis and immune homeostasis, particularly in early B cell development.

## Results

### Patients

**Figure 1.**
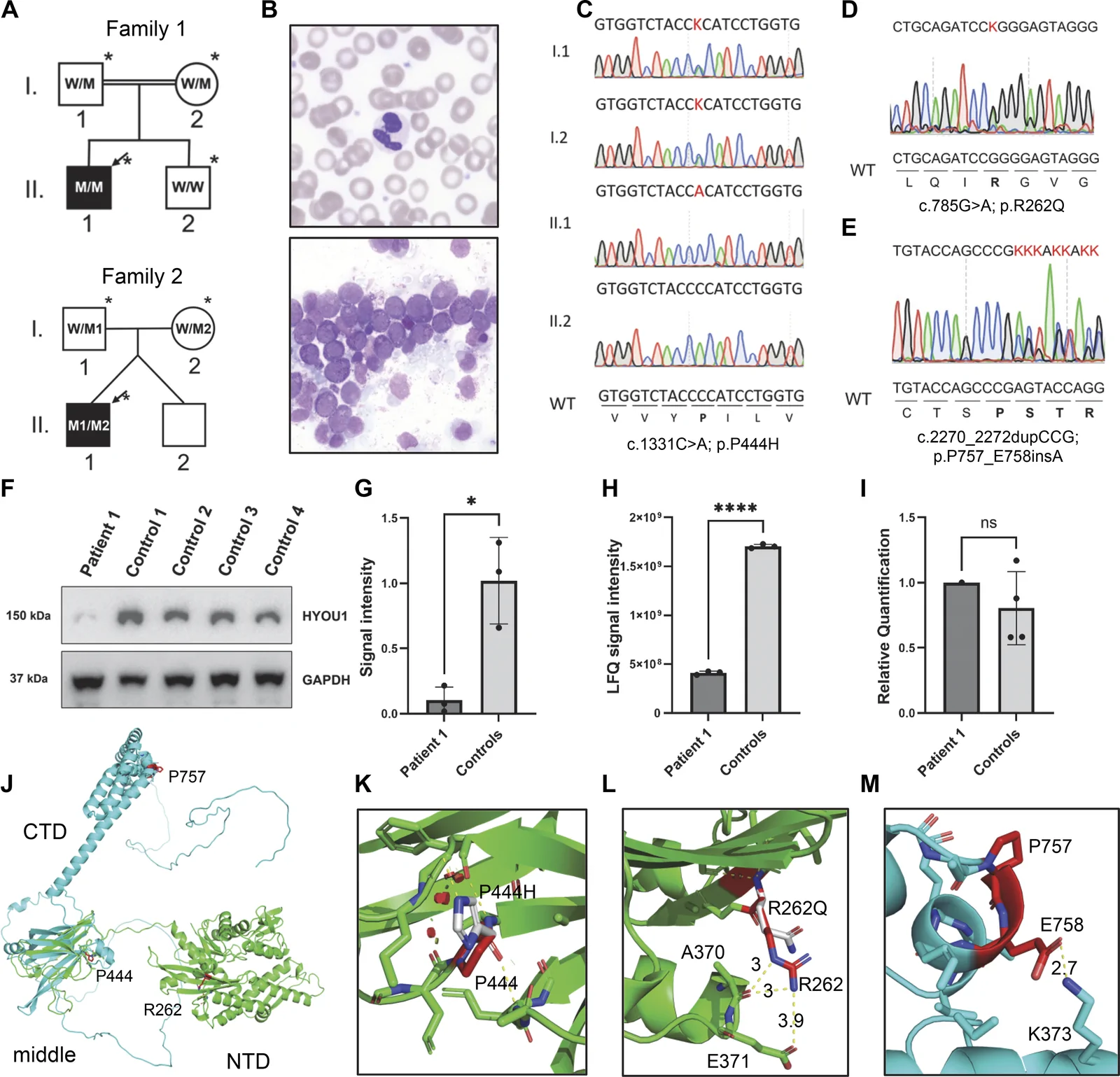
**Genetics, cytology, transcription, translation, and structure of HYOU1 variants in two patients. (A)** Pedigrees of patients. Roman numerals and Arabic numerals indicate generations and subjects, respectively. Double lines connecting the parents connote consanguinity. Squares, male subjects; circles, female subjects; filled (black) symbols represent patients; white symbols represent unaffected family members; arrows indicate the probands in each family; stars show family members subjected to whole-exome sequencing (W: WT allele, M: mutant allele). **(B)** The peripheral blood (upper panel) and bone marrow smears (lower panel) of patient 1 before G-CSF treatment. **(C)** Sanger traces of the *HYOU1* c.1331C>A, p.Pro444His variant in family 1 confirm the homozygous status of the proband and heterozygous status of the parents for the mutation. The electropherograms denote single base pair substitutions of Pro444His in *HYOU1*. **(D)** Sanger traces of *HYOU1* in patient 2 confirm the compound heterozygous status of the patient for the maternally inherited *HYOU1* c.785G>A, p.Arg262Gln variant and **(E)** paternally inherited *HYOU1* c.2270_2272dupCCG>A, p.Pro757_Glu758insAla variant. **(F)** Western blot analyses of HYOU1 protein expression in dermal fibroblasts of patient 1 and four controls. GAPDH was used as the internal control. **(G)** Signal intensity of HYOU1 protein relative to the expression of GAPDH (*P < 0.05). **(H)** Label-free quantification (LFQ) signal intensity of HYOU1 in the proteomics study of the dermal fibroblasts of patient 1 and controls; ****P < 0.0001. **(I)** Quantitative real-time PCR of dermal fibroblasts of patient 1 and controls, normalized to β-actin revealed no difference in *HYOU1* mRNA expression (P = 0.57); ns, not significant. A *t* test was used for statistical comparison. **(J)** Full-length structural modelling of HYOU1 superimposing the 7A4U PDB model (green) and AlphaFold3-predicted structures (blue). Mutated amino acids are indicated in red. **(K–M)** Predicted structural effects of the variants. Wild-type residues are shown in red and mutant residues in gray. Distances between the residues are shown in Å. Source data are available for this figure: SourceData F1.

Patient 1 (Fig. 1 A) was an 8-year-old male of Iranian origin, the first child of consanguineous (first cousins) parents. He was born at early term and small for gestational age (SGA) with a birth weight of 1,900 g (−2.7SD), length of 41 cm (−3.4SD), and head circumference of 29 cm (−3SD). He presented with chronic diarrhea with intermittent exacerbations associated with episodes of hypoglycemia since early infancy, and was evaluated at 6 mo of age for suspected immunodeficiency. At the first investigation, a complete blood count and serum immunoglobulin tests revealed neutropenia (720 cells/μl) and low levels of IgG (123 mg/dl), while IgA and IgM levels were within normal range. IgE was mildly elevated (4 kU/l). Vaccine-specific antibody responses were absent for diphtheria and reduced for tetanus, indicating impaired specific antibody production. On chest X-ray obtained at 6 mo of age, no thymus shadow was observed. Intravenous immunoglobulin (IVIG) replacement therapy was initiated with a provisional diagnosis of agammaglobulinemia. Despite regular IVIG infusions, the patient continued to have several episodes of severe diarrhea and hypoglycemia, as well as recurrent oral aphthae, collectively suggestive of a primary immunodeficiency. Duodenal biopsy at 16 mo of age showed mild duodenitis and a significant decrease in superficial lymphoplasma cells. To correct his neutropenia, granulocyte CSF (G-CSF) (pegylated filgrastim, 100 µg/kg every 14 days) was administered at 3 years 8 mo of age, which clinically resulted in a decrease in the frequency of diarrhea episodes and hospitalizations.

Bone marrow histopathology prior to G-CSF administration indicated a blockade of the granular lineage at the promyelocyte stage (Fig. 1 B, lower panels) with the presence of a few myeloblasts, the absence of myelocytes and metamyelocytes, rare hypogranulated polymorphonuclear neutrophils, and an increase in monocytes. The erythroid lineage was relatively diminished but with normal maturation. Following G-CSF, bone marrow examination showed a non-blastic–rich marrow, alongside a predominance of the granular lineage without maturation block and hypogranularity of neutrophils with nuclear segmentation anomalies, particularly hyposegmentation (Fig. 1 B, upper panel). At the last evaluation at 4 years and 6 mo of age, his weight was 8,500 g (−8.2 SD), height 80 cm (−5.7SD), and head circumference 43 cm (−5.3 SD), with minor facial dysmorphism. He met neurodevelopmental milestones for his age. Additional laboratory results are shown in Table S1. At present, he is being treated with monthly IVIG, peg-filgrastim, and prophylactic cotrimoxazole.

Patient 2 was a 32-year-old male born to healthy non-consanguineous parents, with a healthy fraternal twin brother, normal birth weight, and normal growth and development (Fig. 1 A). He presented for >10 years with a history of immune deficiency with associated enteropathy, fatigue, and portal hypertension. He had a long history of recurrent infections, including middle ear, upper respiratory tract, and recurrent viral and bacterial pneumonia, as well as chronic lymphadenopathy requiring adenectomy at an early age. From his late teenage years, he has had several episodes of chronic diarrhea without hematochezia or melena and was diagnosed with common variable immunodeficiency (CVID) in his mid-20s, for which IVIG therapy was initiated. Subsequently, he developed skin rashes, recurrent leg swelling, chronic extreme fatigue, and progressive osteoporosis. Endoscopy noted diffuse mildly scalloped mucosa in both D1 and D2 regions, with follow-up biopsies demonstrating mildly active chronic duodenitis with mild focal atrophy and intraepithelial lymphocytosis. Prior targeted celiac disease gene testing was negative. Furthermore, the patient did not report any episodes of hypoglycemia as an adult; however, his anamnesis had documented the need for a late-night snack as a child, as well as the fact that he never skipped breakfast. Laboratory workup showed CVID (Table S1).

### Both patients carried recessive variants in *HYOU1*

Exome sequencing was performed for patient 1, his healthy brother, and both parents. Applying successive filtering steps based on variants’ functional consequences, frequency, and autosomal recessive inheritance, we identified a missense variant, c.1331C>A (GenBank accession no. NM_001130991.3); p.Pro444His (GenBank accession no. NP_001124463.1) in the *HYOU1* gene that is predicted to be deleterious by various bioinformatic prediction tools (Table 1). The remaining candidate variants and their annotation are shown in Table S2. Sanger sequencing confirmed the homozygous status of this variant in the patient, its heterozygous status in his parents, and the WT homozygous status in his brother (Fig. 1 C).

**Table 1.** Candidate variant annotations and predicted impact in patients

Patient 1 variant	
Genomic position (hg38)	chr11: 119051826
Gene symbol	*HYOU1*
HGVSc: Nucleotide change	NM_001130991.3: c.1331C>A
HGVSp: Protein change	NP_001124463.1: p.Pro444His
gnomAD frequency	1.24.10^−6^ heterozygotes (no homozygotes)
PolyPhen (score)^a^	Probably damaging
CADD (phred score)^b^	16.5
GERP++_RS^c^	4.96
MutationTaster	Disease causing
PhastCons^d^	0.83
**Patient 2 variant 1**	
Genomic position (hg38)	chr11: 119054130
Gene symbol	*HYOU1*
HGVSc: Nucleotide change	NM_001130991.3: c.785G>A
HGVSp: Protein change	p. Arg262Gln
gnomAD frequency	4.458.10^−5^
PolyPhen (score)^a^	Benign
CADD (phred score)^b^	25.3
GERP++_RS^c^	3.55
MutationTaster	Deleterious
PhastCons^d^	0.802
**Patient 2 variant 2**	
Genomic position (hg38)	chr11: 119048352
Gene symbol	*HYOU1*
HGVSc: Nucleotide change	NM_001130991.3: c.2270_2272dup
HGVSp: Protein change	p. Pro757_Glu758insAla
gnomAD frequency	Absent
PolyPhen (score)^a^	Probably damaging
CADD (phred score)^b^	42
GERP++_RS^c^	5.54
MutationTaster	Deleterious
PhastCons^d^	0.869

a Polyphen2_HDIV_score: variants with scores between 0.85 and 1.0 are predicted to be damaging with high confidence.

b CADDv1.4 scores range from 1 to 99, with a higher score indicating greater deleteriousness.

c GERP++_RS score: DNA conservation score. Scores range from 1 to 6.18. The larger the score, the more conserved the site.

d PhastCons score: probability of negative selection and ranges between 0 and 1.

The DNA of the patient 2 was submitted for clinical exome sequencing along with parental DNA for segregation, revealing biallelic variants in *HYOU1*. A maternally inherited missense variant c.785G>A (GenBank accession no. NM_001130991.3); p.Arg262Gln (GenBank accession no. NP_001124463.1) (Fig. 1 D) and a paternally inherited insertion c.2270_2272dupCCG (GenBank accession no. NM_001130991.3); p.Pro757_Glu758insAla (GenBank accession no. NP_001124463.1) (Fig. 1 E). Both variants were predicted to be damaging by in silico prediction tools (Table 1) and were rare (frequency of 4.84 × 10^−5^ for p.Arg262Gln) or absent (p.Pro757_Glu758insAla) in the Genome Aggregation Database (gnomAD), with the substitution occurring in the ATPase domain and the insertion in the α-helical lid (11).

All three variants affected evolutionarily constrained positions, as evidenced by high GERP++ RS scores (4.96, 3.55, and 5.54, respectively) and PhastCons scores approaching 1 (0.83, 0.802, and 0.869, respectively), supporting their functional significance (Table 1).

### The Pro444His variant did not affect mRNA levels but drastically diminished protein levels

Western blotting indicated a significant decrease in the HYOU1 protein level as compared with controls (Fig. 1 F). Quantification of these results showed that the HYOU1 protein level in patient 1 represented ∼10% of the protein level in controls (P < 0.05, Fig. 1 G). This finding was in line with the results of proteomic analysis, in which label-free quantification intensity of HYOU1 compared with controls was ∼20% (P <0.0001, Fig. 1 H). Quantitative RT-PCR revealed no significant difference in the expression of the *HYOU1* transcript in the patient 1 compared with controls (Fig. 1 I). Hence, the variant does not affect transcription, yet drastically diminishes the amount of detectable protein.

### The *HYOU1* variants were predicted to perturb the structure of the protein

**Figure S1.**
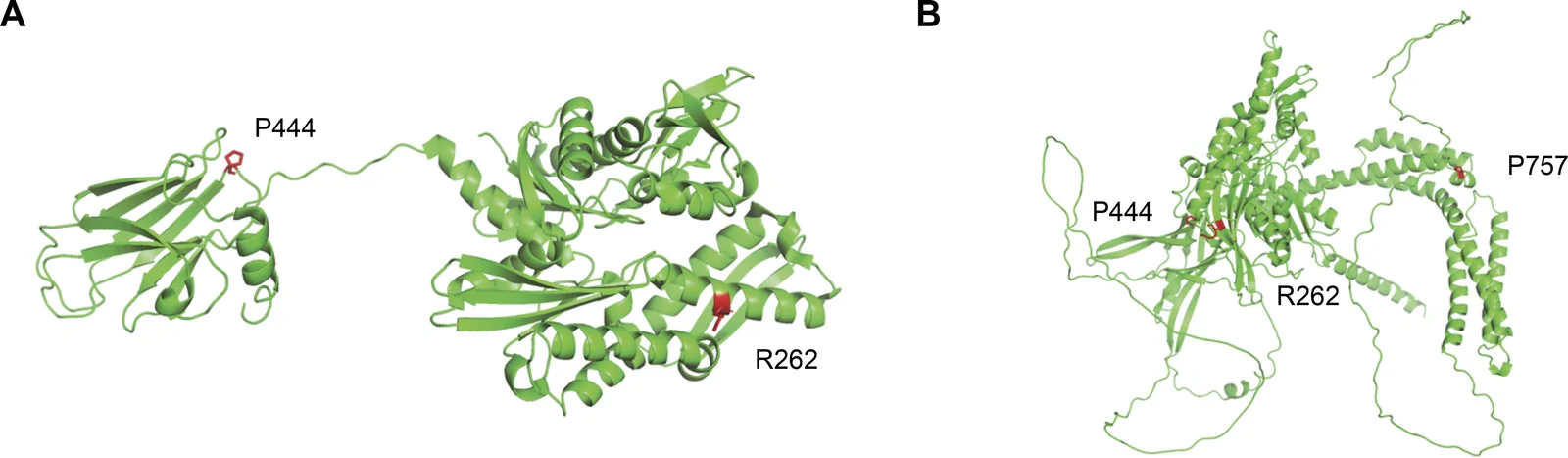
**Structural modelling of HYOU1 with 7A4U template in SWISS-MODEL. (A)** and using AlphaFold3. **(B)** P444, R262, and P757 (red) are located in regular secondary structure regions.

To evaluate the structural consequences of the three *HYOU1* variants, we first assessed the reliability of available structural templates. No full-length structural homologues could be identified; however, the N-terminal domain (NTD) aligned closely with the PDB entry 7A4U, which provided a high-quality template for homology modelling (Fig. S1 A). In contrast, an end-to-end prediction using AlphaFold3 produced a markedly different global topology, with extensive low-confidence and unstructured regions that did not conform to the 7A4U-derived architecture (Fig. S1 B). Although we therefore did not use the full AlphaFold3 model for structural presentation, it is notable that all three variants localize within predicted ordered secondary-structure elements rather than disordered segments. To obtain a more coherent full-length model, we combined the 7A4U-based NTD with the AlphaFold3-predicted middle and C-terminal domains (CTDs), using the central domain as an anchor to guide domain placement. This hybrid approach yielded good agreement between the experimentally derived and predicted regions, with the middle domain aligning to the template with an RMSD of ∼2 Å, supporting the overall structural plausibility of the model (Fig. 1 J).

Mapping the variants onto this model suggests plausible structural perturbations. The p.Pro444His substitution affects a residue that caps a β-strand: Pro444 contributes to the backbone hydrogen bonds defining the β-sheet boundary, and its replacement with His may both extend the β-strand and introduce steric clashes because of the bulkier imidazole ring (Fig. 1 K). Arg262 forms stabilizing hydrogen bonds with the backbone oxygen of Ala370 and the side chain of Glu371; the substitution to Gln shortens the side chain and abolishes these interactions, potentially destabilizing the helical terminus (Fig. 1 L). Finally, the p.Pro757_Glu758insAla insertion disrupts a helix-terminating Pro residue and may interfere with a stabilizing Glu758–Lys373 hydrogen bond, thereby compromising local helix integrity (Fig. 1 M). Together, these structural considerations support the functional relevance of the three *HYOU1* variants.

### A sharp decrease in B cells and perturbations in T and NK cells

**Figure 2.**
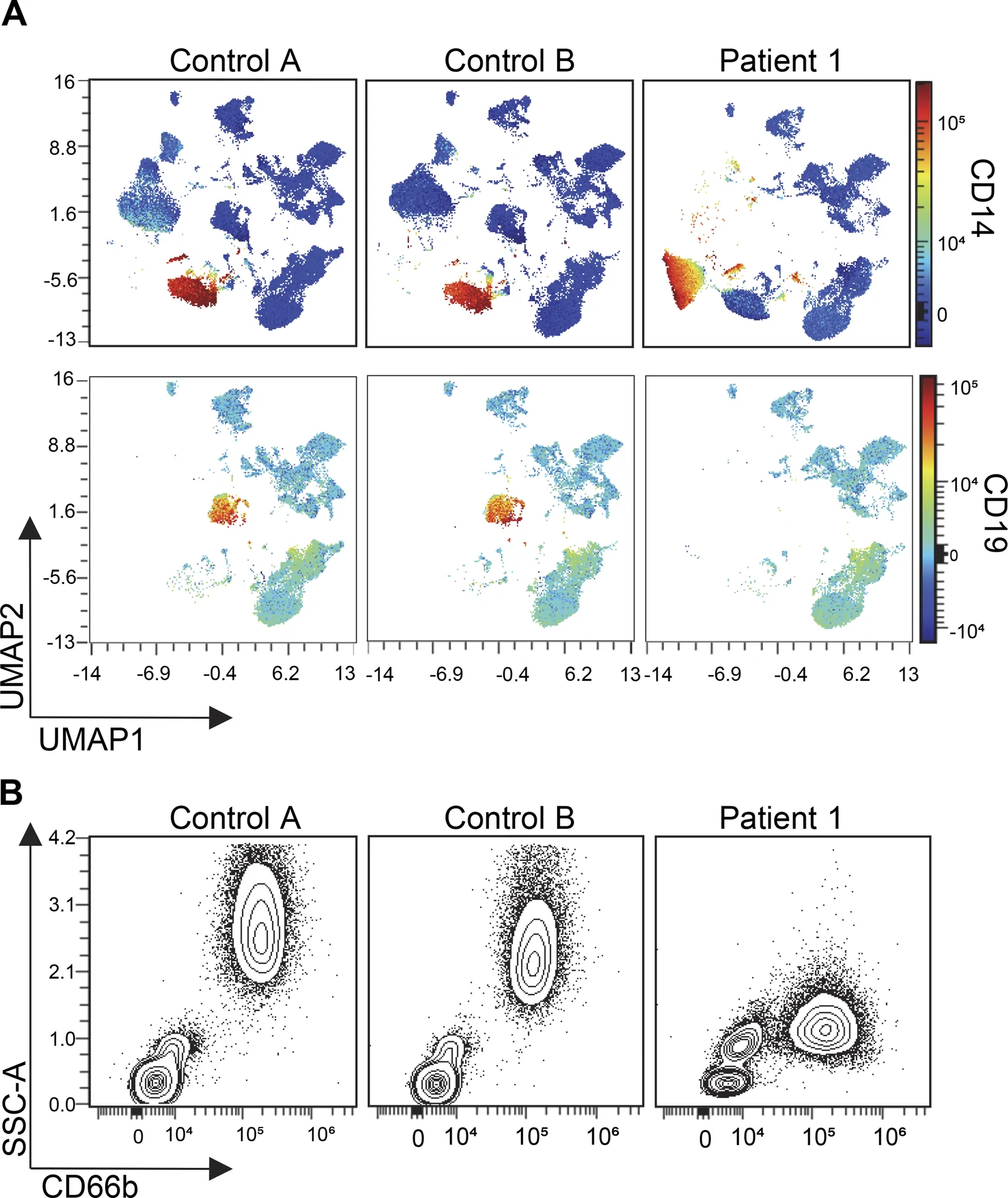
**A depiction of flow cytometry experiments in patient 1. (A)** The UMAP visualization depicts the analysis of white blood cells obtained from flow cytometry data of patient 1 and 2 controls, colored based on expression levels of CD14 and CD19. The granulocytes of patient 1 are different in the expression of CD14. Almost no CD19^+^ B cells are observed in patient 1. **(B)** Flow cytometry scatter plot depicting granulocytes in patient 1 and 2 controls, labelled with CD66b on the x-axis and side scatter (SSC) on the y-axis. The patient’s granulocytes have considerably lower SSC-A, indicating lower granularity.

Compared with age-matched controls, immunophenotyping revealed a significant decrease in the B cell compartment (CD19^+^) in patient 1 (0.14 and 0.2% detected in two different samplings of leukocytes of the patient; normal range 10.21–20.12%) (Table 2 and Fig. 2 A). All B cell subsets were drastically reduced (Table 2). T cell abnormalities were also present, characterized by a shift toward terminally differentiated effector memory subsets within both CD4^+^ and CD8^+^ compartments, at the expense of naive and central memory populations, along with decreased CD279 (PD1) expression and increased CD57 expression indicative of senescence. CD4^+^ follicular helper T cells (CD4^+^CXCR5^+^) were profoundly reduced, and the percentage of γδ T cells was decreased. Mature NK cells (CD56^dim^CD16^+^) were decreased, with a relative increase in CD56^bright^ subsets. Although the number and percentage of the patient’s neutrophils were normal (following the administration of G-CSF), we observed two additional atypical populations in the granulocytes of the patient (Fig. 2, A and B), characterized by significantly lower-than-expected granules (SSC^lo^). An increase in the percentage of plasmacytoid dendritic cells (CD123^+^CD11c^−^) was also observed in the patient compared with controls. Full subset data are provided in Table 2.

**Table 2.** Hematological and Immunological parameters of patient 1 at 5 years of age

​	Sample 1^a^	Sample 2^a^	Normal range^b^
Red blood cells (10^6^ cells/μl)^1^	5.15	​	3.9–5.3
Hemoglobin (g/dl)^1^	11.4	8.4	11.5–14.5
Hematocrit (%)^1^	38	26.8	34–40
Mean corpuscular volume (fL)^1^	73.8	75.7	76–90
Mean corpuscular hemoglobin (pg)^1^	22.1	23.7	25–30
Mean corpuscular HGB (g/dl)^1^	30	31.3	32–36
Red cell distribution width-CV (%)^1^	26.5	22.4	11.5–15
Platelet count (10^3^/μl)^1^	235	144	150–450
White blood cells (10^3^ cells/μl)^1^	9.3	7.23	5–14.5
**Granulocytes**	​	​	​
Granulocytes (%)^1^	41	44.3	​
Neutrophils (10^3^ cells/μl)^1^	3.72	3.19	1.5–8
Neutrophils (%)^3^	40	44.2	28–54
Basophils (10^3^ cells/μl)^3^	​	0.01	0–0.2
Basophils (%)^1^	​	0.1	0–1
Eosinophils (10^3^ cells/μl)^3^	0.93	0	0–0.7
Eosinophils (%)^1^	1	0	0–3
**Monocytes**	​	​	​
Monocytes (10^3^ cells/μl)^3^	0.372	1.28	0.1–1.5
Monocytes (%)^1^	4	17.7	0–5
**Lymphocytes by CBC**	​	​	​
Lymphocyte (cells/μl)^2^	5,115	2,750	2,280–3,820
Lymphocyte (%)^1^	55	38	17–67
**Immunophenotyping**	​	​	​
CD3^+^ (cells/μl)^2^	3,313	3,703	1,424–2,664
CD3^+^ (%)^2^	84.84	92	60.05–74.8
CD56^+^ (cells/μl)^2^	466	326	​
CD56^+^ (%)^2^	12.73	8.2	​
CD4^+^ (cells/μl)^2^	1,694	2,158	686–1,358
CD4^+^ (%)^2^	43.49	54.2	26.17–40.76
CD8^+^ (cells/μl)^2^	1,440	1,353	518–1,125
CD8^+^ (%)^2^	36.88	34	19.68–34.06
CD4/CD8 ratio^2^	1.17	1.6	0.87–1.94
CD4^+^CD8^+3^	0.26	0.14	0–1
CD19^+^ (cells/μl)^2^	5	6	280–623
CD19^+^ (%)^2^	0.14	0.2	10.21–20.12
**% in CD3^+^ T cells**	​	​	​
CD3^+^TcRγδ^−^ (%)^2^	96.03	96.34	90.66–93.08
CD3^+^TcRγδ^+^ (%)^2^	3.97	3.66	6.92–9.34
**% in CD4** ^+^ **T cells**	​	​	​
Naive (CD45RA^+^CCR7^+^)^2^	78.3	77.7	45.56–75.28
Central memory (CD45RA^−^CCR7^+^)^2^	6.2	6.9	22.06–46.46
Effector memory (CD45RA^−^CCR7^−^)^2^	13.2	12.7	2.08–8.78
CD45RA^+^ TEMRA (CD45RA^+^CCR7^−^)^2^	2.2	3.7	0–1.06
Treg (CD25^+^CD127^−^)	5.9	6.2	5.81–10.68
PD1^+^ (CD279^+^)^3^	4.6	1	14–32
Follicular helper (CXCR5^+^)	0.5	0.8	6.5–10.1
**% in CD8^+^ T cells**	​	​	​
Naïve (CD45RA^+^CCR7^+^)^2^	39.7	32.9	41.58–77.90
Central memory (CD45RA^−^CCR7^+^)^2^	0.2	0.8	12.08–30.54
Effector memory (CD45RA^−^CCR7^−^)^2^	11.1	13.2	1.58–13.18
CD45RA^+^ TEMRA (CD45RA^+^CCR7^−^)^2^	48.8	51.6	1.70–24.62
PD1^+^ (CD279^+^)^3^	4.5	0.6	15–47
CD57^+3^	23.3	32.9	6–32
**% in CD19^+^CD20** ^+^ **B cells**	​	​	​
Switched memory (IgD^−^CD27^+^)^2^	Not detectable	Not detectable	7.76–19.9
Unswitched memory (IgD^+^CD27^+^)^3^	Not detectable	Not detectable	5–20
Naive (IgD^+^CD27^−^)^2^	Not detectable	Not detectable	48.36–75.84
**% in monocytes**	​	​	​
Classical (CD14^+^CD16^−^)^3^	71.4	57	69–87
Transient (CD14^+^CD16^+^)^3^	2	1.6	2–19
Resident (CD14^lo^CD16^+^)^3^	0.5	6.8	2–8
**% in lineage** ^**−**^ **HLA DR** ^**+3**^	​	​	​
Plasmacytoid dendritic cells CD123^+^	50.27	54.67	4.38–8.98
**% in CD16** ^+^ **CD56** ^+^ **NK cells**	​	​	​
CD56^bright^CD16^−^ (CD56^+^)^3^	12.8	8.6	1–12
CD56^bright^CD16^+^ (CD56^++^CD16^+^)^3^	77.5	58.6	0–6
CD56^dim^/CD16^+^ (CD56^+^CD16^+^)^3^	9.6	32.8	72–96
NKG2C^+^ (CD94^+^)^3^	8.2	7.1	3–23

TEMRA, terminally differentiated effector memory subset.

a Samples 1 and 2 were taken 2 mo apart.

b Normal range values are either from (1) Gregory and Andropoulos (12), https://doi.org/10.1002/9781444345186, (2) Ding et al. (13), https://doi.org/10.1016/j.jaci.2018.04.022, or (3) the onsite clinical laboratory.

### A reduced response to ER stress

**Figure 3.**
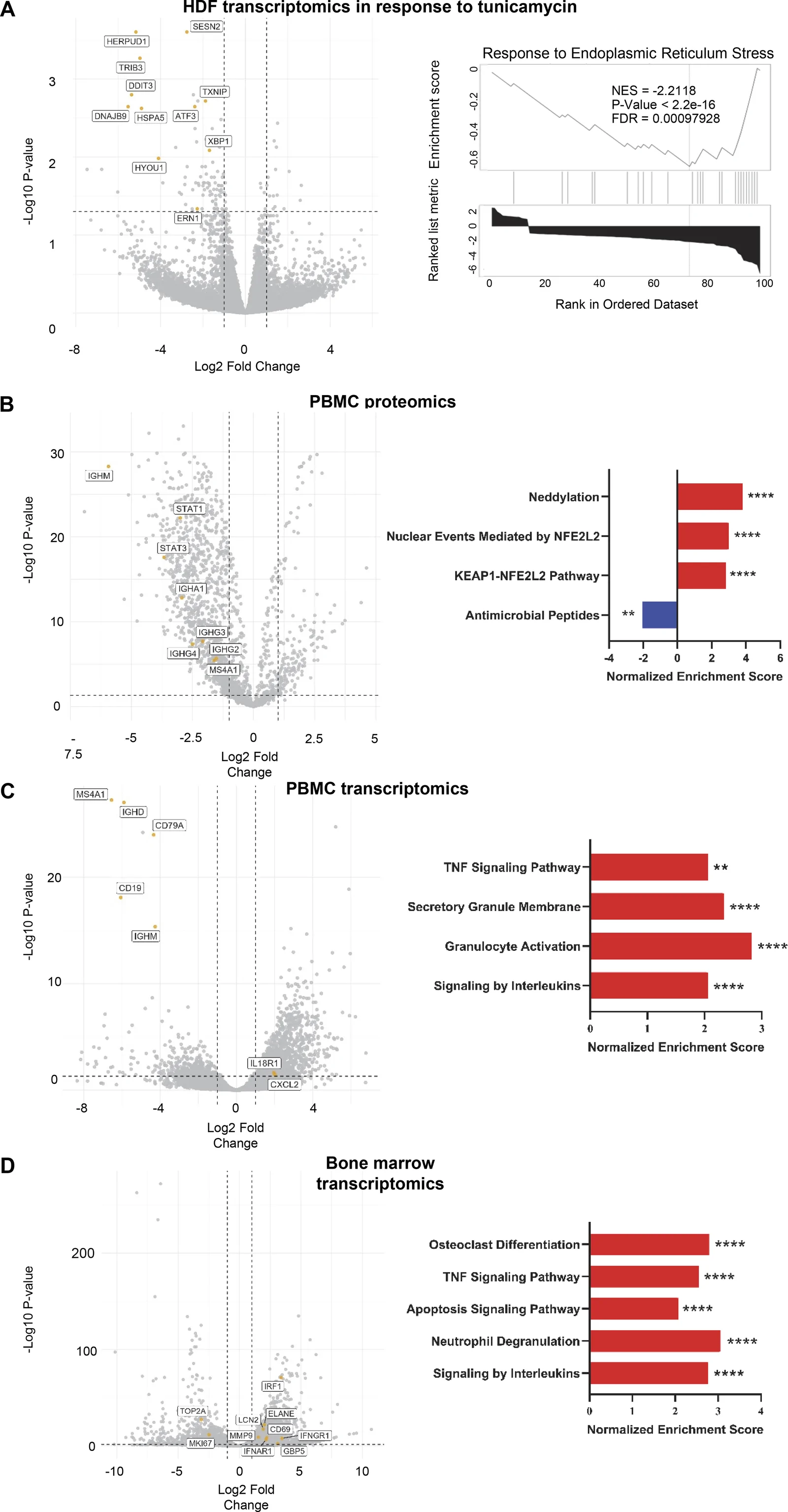
**Multi-omics analyses of patient 1 cells compared with controls under normal and stress conditions. (A)** Transcriptomic analysis in HDF in response to tunicamycin treatment compared with the controls and normalized to mock treatment. **(B–D)** Proteomic and transcriptomic analyses of PBMC (B and C), and transcriptomic analyses of bone marrow samples (D). Left panels show volcano plots demonstrating up- and downregulated genes and proteins in patient 1 compared with the controls, respectively, using log2 fold change and P value cutoffs of 1 and 0.05, respectively. Right panels show dysregulated canonical pathways in patient 1 compared with controls; **P < 0.01; ****P < 0.0001. GSEA was performed in WebGestalt using a weighted Kolmogorov–Smirnov statistic, with obtained P values from permutation testing and adjusted using FDR (Benjamini–Hochberg). FDR, false discovery rate; NES, normalized enrichment score.

To compare ER stress and its regulatory mechanisms such as UPR, we performed RNA-seq on dermal fibroblasts of patient 1 and healthy controls, treated or not with tunicamycin. After 6 h of exposure to tunicamycin, we compared the treatment effect in the patient with the treatment effect in the controls, normalizing the response to mock treatment. We observed that UPR genes (*HSPA5* [BiP/GRP78]*, XBP1,* and *ERN1* [IRE1α]), and *HYOU1* were less induced in tunicamycin-treated cells of the patient compared with controls (Fig. 3 A). The majority of significantly less induced genes in the patient were ER stress regulator genes such as *DNAJB9* (ERdj4), *TRIB3*, *DDIT3* (CHOP), *TXNIP*, and *HERPUD1* (HERP). Sestrin-2 (*SESN2*), related to reactive oxygen species, was among the genes significantly less induced (Fig. 3 A). The UPR inducer *ATF3* was also significantly less induced in the patient. Finally, gene set enrichment analysis (GSEA) revealed that the response to ER stress process was significantly less induced in the patient versus the controls after stress induction (Fig. 3 A).

**Figure S2.**
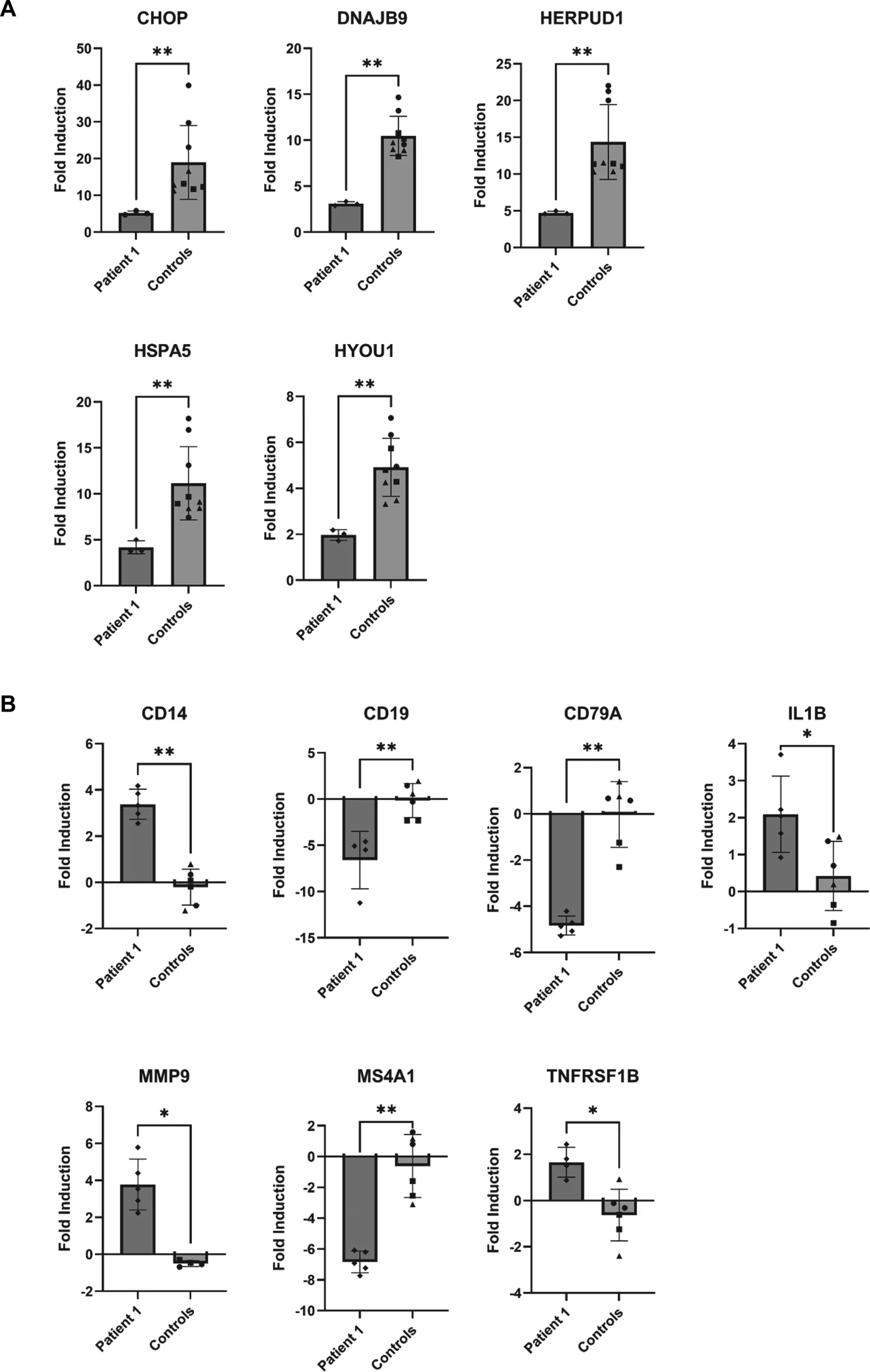
**Quantitative real-time PCR analysis of cells from patient 1 and healthy controls. (A)** Dermal fibroblasts (HDFs) from patient 1 and three healthy controls were treated with tunicamycin (0.5 µg/ml) or DMSO as vehicle control for 6 h prior to RNA extraction. Relative expression levels were calculated using the ΔΔCt method after normalization to the geometric mean of GAPDH and HPRT1 and expressed as fold induction. Data are shown as individual biological replicates. **(B)** PBMCs from patient 1 and three healthy controls. Relative expression levels were calculated using the ΔΔCt method after normalization to the geometric mean of GAPDH and HPRT1 and are presented as log2 fold change (log2FC). For both panels, statistical comparisons between patient 1 and controls were performed using two-sided unpaired Student’s *t* tests followed by Benjamini–Hochberg correction for multiple testing. Statistical significance is indicated as follows: *P < 0.05; **P < 0.01; ns, not significant.

**Figure S3.**
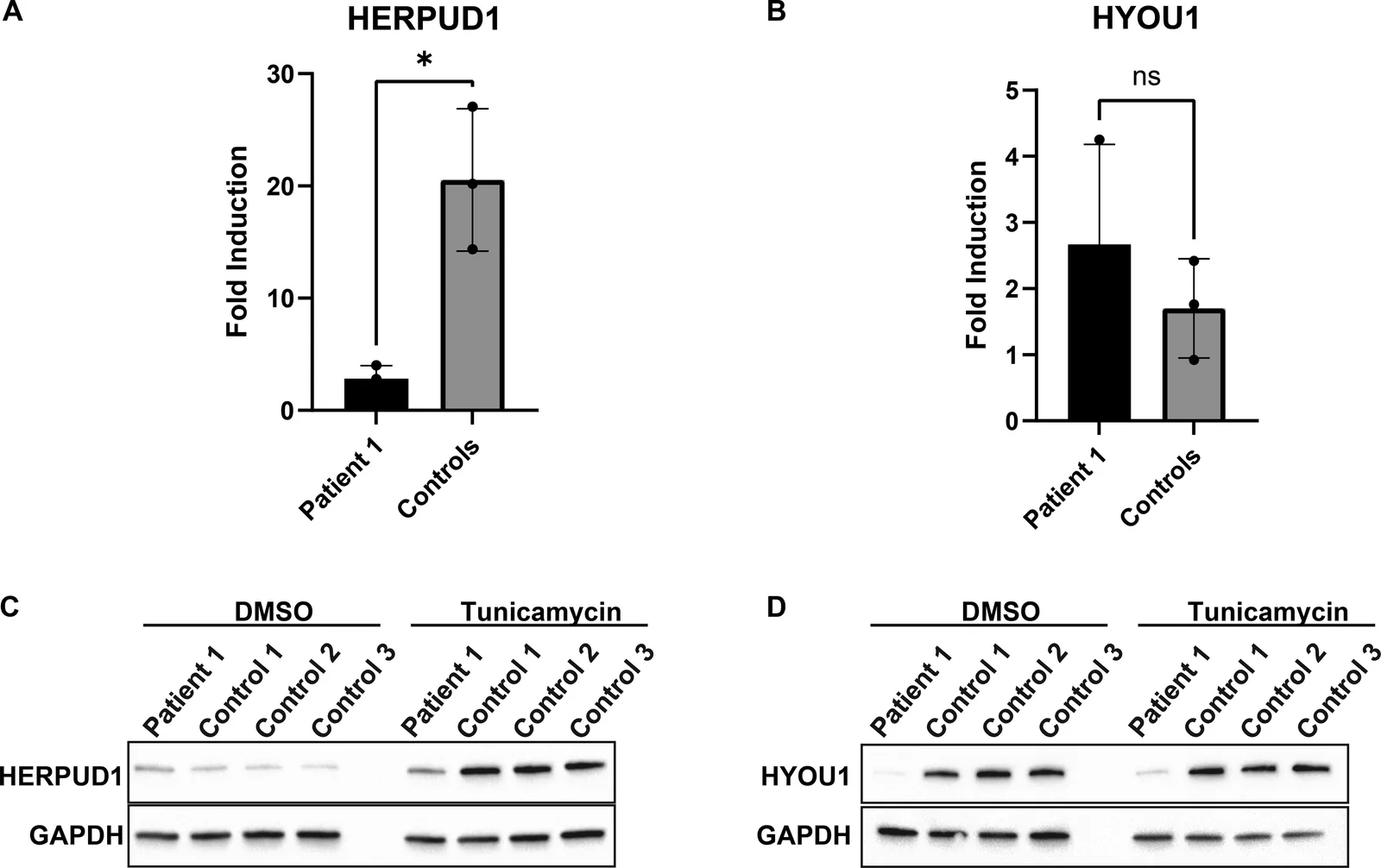
**Western blot analyses of tunicamycin-treated dermal fibroblasts from patient 1 and three healthy controls.** HDFs were treated with tunicamycin (0.5 µg/ml) or DMSO as a vehicle control for 6 h prior to RNA extraction. **(A–D)** Relative HERPUD1 (A–C) or HYOU1 (B–D) protein expression levels were normalized to GAPDH and expressed as fold induction. Statistical comparisons were performed using two-sided unpaired Welch’s *t* tests. Data are shown as individual biological replicates. Statistical significance is indicated as follows: *P < 0.05; ns, not significant. Source data are available for this figure: SourceData FS3.

To validate these transcriptomic findings by an independent method, we performed RT-qPCR on tunicamycin-treated human dermal fibroblasts (HDFs) from patient 1 and three healthy controls. Consistent with the RNA-seq results, qPCR confirmed that fold induction of *HYOU1*, *HSPA5* (BiP/GRP78), *DNAJB9* (ERdj4), *HERPUD1* (HERP), and *DDIT3* (CHOP) was significantly reduced in the patient relative to pooled controls (Fig. S2). Western blot analysis further confirmed that HERPUD1 protein induction following tunicamycin treatment was significantly reduced in the patient compared with pooled controls (P < 0.05, Fig. S3, A–C). For HYOU1, fold induction at the protein level was not significantly different between the patient and controls (Fig. S3 B); however, given that the baseline HYOU1 protein level in the patient was ∼10% of that in controls (Fig. 1 G), the absolute induced protein level remained markedly lower than in controls despite comparable fold induction (Fig. S3 D).

### Dysfunctionality in B cell and innate immune compartments

Differential expression analyses were performed on peripheral blood mononuclear cell (PBMC) transcriptomics and proteomics data of patient 1. The most downregulated genes observed in the transcriptomics data were B cell marker genes and genes involved in B cell activation (*MS4A1*, *CD19*, *IGHD*, *CD79A*, and *IGHM*). Similarly, proteomics analysis revealed a drastic downregulation of B cell–related proteins (MS4A1, IGHM, IGHA1, IGHG2, IGHG3, and IGHG4), reflecting the B cell deficiency in patient 1 (Fig. 3, B and C). In proteomics, neddylation, nuclear events mediated by NF2L2, and KEAP1–NFE2L2 pathways were enriched in the patient (Fig. 3 B). In transcriptomics, we noticed the alteration of certain innate immunity pathways. Among these, the TNF-signalling pathway, signalling by interleukins, secretory granule membrane, and granulocyte activation were upregulated in the patient compared with controls (Fig. 3 C), while in proteomics data, the antimicrobial peptide pathway was negatively enriched in the patient (Fig. 3 B). We also observed downregulation of the STAT1 and STAT3 proteins, signal transducers for G-CSF, in the proteomics data (Fig. 3 B). In transcriptomics, *IL18R1* was significantly upregulated in the patient in comparison with the control, which indicates the activation of IFNγ-regulated cytokines, and the key neutrophil attractant *CXCL2* was significantly upregulated in the patient (Fig. 3 C).

To validate these findings, we performed RT-qPCR on archived PBMC pellets from patient 1 and three related controls (father, mother, and brother of the patient, who are heterozygous carriers). B cell marker genes *CD79A* and *MS4A1* were significantly reduced in the patient relative to family controls, consistent with the B cell deficiency observed by transcriptomics and flow cytometry (Fig. S2 B). *CD14* expression was significantly elevated in the patient compared with his father and mother, in line with the expansion of monocyte populations identified by immunophenotyping. Among innate immune markers, *IL1B*, *MMP9*, and *TNFRSF1B* were significantly upregulated in the patient compared with family controls, corroborating the transcriptomic findings of enriched signalling by interleukins, secretory granule membrane, and TNF signalling pathways in the patient (Fig. S2 B).

### Abnormal IFN response

In bulk transcriptomics analysis of bone marrow cells of patient 1 compared with control, certain genes associated with IFN response (*IRF1*, *GBP5*, and *CD69*) were upregulated, as well as genes encoding subunits of IFNβ and IFNγ receptors (*IFNAR1*, *IFNGR1*). Genes encoding proteins in azurophilic granules, gelatinase granules, and specific granules (*ELANE*, *MMP9*, and *LCN2*, respectively) were upregulated, while the cell cycle genes (*MKI67*, *TOP2A*) were downregulated (Fig. 3 D). The apoptosis and neutrophil degranulation signalling pathways were upregulated in the patient in comparison with the controls (Fig. 3 D). Moreover, the TNF-signalling pathway was upregulated in the patient in conformity with the findings of the RNA sequencing in the PBMC (Fig. 3 C). In line with the failure to thrive (FTT) observed in the patient, osteoclast differentiation was upregulated (14) (Fig. 3 D).

### Neutrophil dysfunction

**Figure 4.**
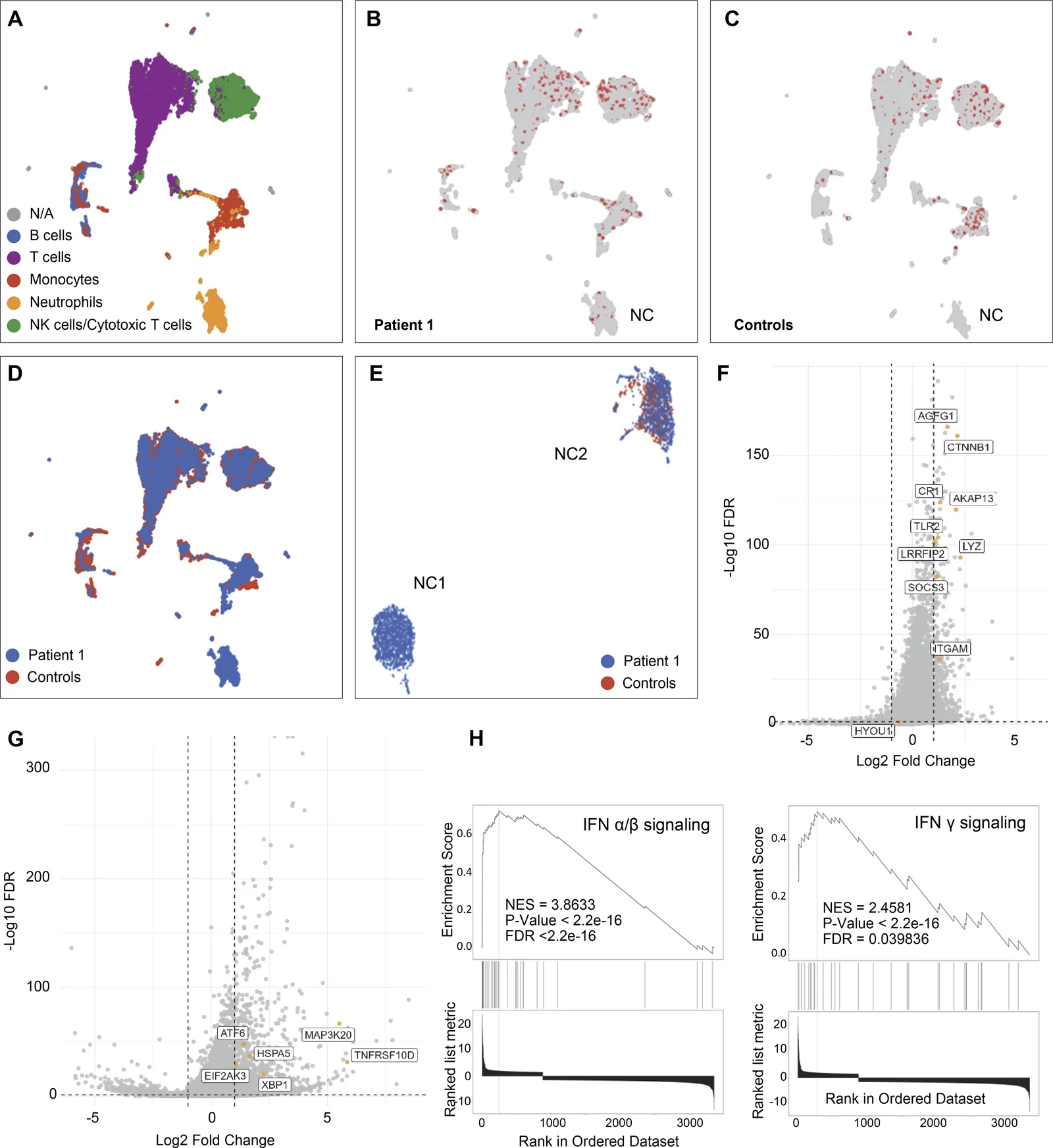
**scRNA-seq analyses of blood cells. (A)** UMAP plot showing blood cells colored by cell type. **(B and C)** UMAP plot showing HYOU1 normalized counts for the patient (B) and the controls (C) in blood as red dots (NC, neutrophil clusters). **(D)** UMAP plot showing blood cells for the patient and controls; colors correspond to group. **(E)** Reclustering of blood neutrophils shown in UMAP plot (blue, patient cells; red, control cells). **(F and G)** Volcano plots showing the differentially expressed genes between neutrophil cluster 1 and cluster 2, and between patients’ neutrophils and controls’ neutrophils, respectively. **(H)** GS EA plots displaying the enrichment of significantly upregulated pathways IFNα/β signalling and IFNγ signalling pathways in NC1 compared with NC2. FDR, false discovery rate; NES, normalized enrichment score. Analysis was performed in WebGestalt using weighted Kolmogorov–Smirnov statistic, with obtained P values from permutation testing and adjusted using FDR (Benjamini–Hochberg).

**Figure S4.**
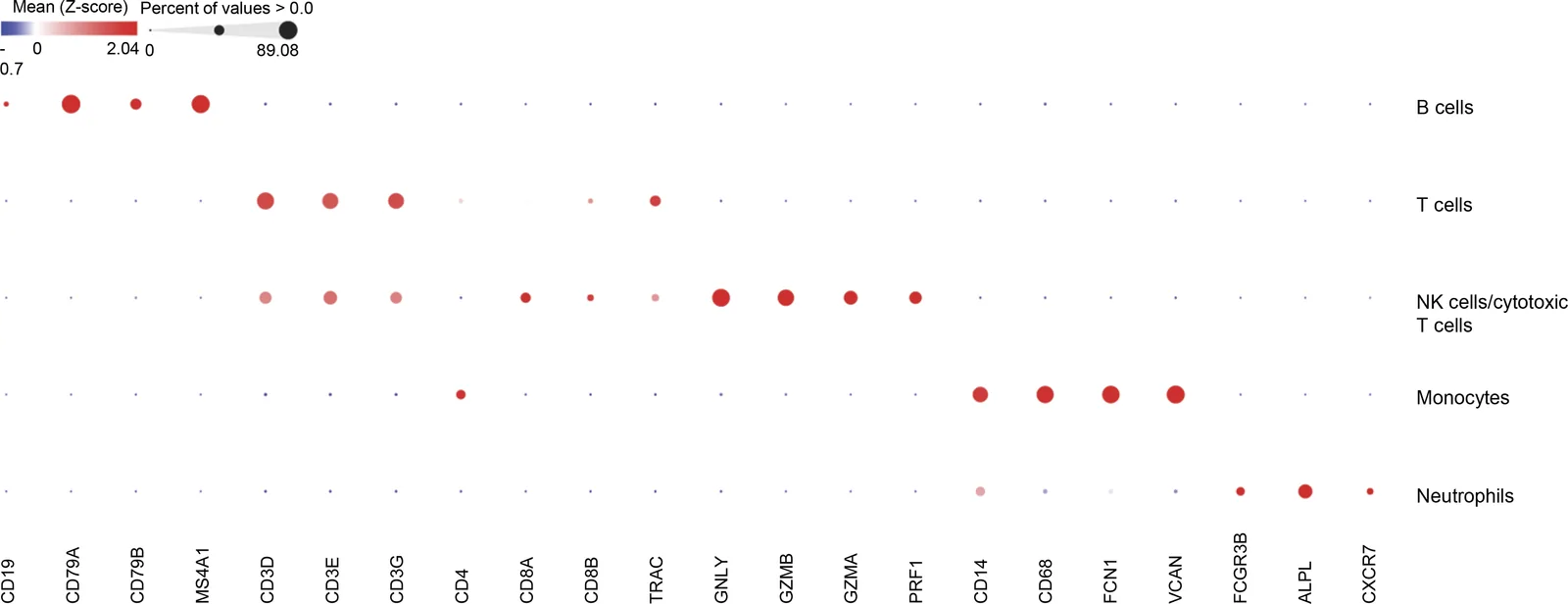
**Normalized expression of canonical gene markers demonstrated in bubble map plots according to the annotated cell type**.

We performed single-cell RNA sequencing (scRNA-seq) on unsorted cells obtained from peripheral blood and bone marrow samples of patient 1 and healthy controls. Following quality control and normalization, 28,757 cells were obtained in total in blood samples (Fig. 4 A; *n* = 15,737 composed of three biological replicates for patient 1 and *n* = 13,021 cells in the four controls). After performing principal component (PC) analysis (PCA) for the screening of significantly correlated genes, we selected 20 PCs for further analysis. We visualized the cells using Uniform Manifold Approximation and Projection (UMAP). We used the following markers to identify the clusters: *CD19*, *CD79A*, *CD79B*, and *MS4A1* for B cells, *CD3D*, *CD3E*, *CD3G*, *CD4*, *CD8A*, *CD8B*, and *TRAC* for T cells, *GNLY*,* GZMB*, *GZMA*, and *PRF1* for NK cells and cytotoxic cells, *CD14*, *CD68*, *FCN1*, and *VCAN* for monocytes, and *FCGR3B*, *ALPL*, and *CXCR2* for neutrophils (Fig. S4). *HYOU1* was expressed in all clusters of patient 1 and controls, except in the controls’ neutrophils (Fig. 4, B and C).

There was a drastic reduction of B cells in the patient (*n* = 0 for a total of three biological replicates) compared with the controls (*n* = 2,541 for a total of four controls, Fig. 4, A and D). In addition, we observed that the majority of the neutrophil and monocyte populations were from the patient (patient = 5,326, controls = 1,381), which could be due to treatment with G-CSF (Fig. 4, A and D). A more in-depth analysis of neutrophils showed two completely distinct clusters of neutrophils in patient 1 and controls (Fig. 4 E). One cluster was composed solely of the patient’s neutrophils (neutrophil cluster 1, NC1), whereas the other was composed of both the patient’s and controls’ neutrophils (neutrophil cluster 2, NC2). Further comparing NC1 to NC2 (Fig. 4 F), we noticed that gelatinase (*LYZ*) and secretory (*CR1* and *ITGAM*) granule genes were overexpressed. Additionally, apoptotic-related genes *AGFG1* and *CTNNB1* and the death receptor signalling gene *AKAP13* were upregulated*. TLR2* and *LRRFIP2,* encoding a protein involved in toll-like receptor signalling, were also overexpressed in the NC1. The expression of *HYOU1* was not different between the two neutrophil clusters (Fig. 4 F). Additionally, we compared the neutrophils of the patient with those of the controls (Fig. 4 G). We observed that *ATF6* (ATF6α), *XBP1*, *EIF2AK3* (PERK), and *HSPA5* (BiP/GRP78), encoding proteins crucial in the UPR, are overexpressed in this comparison. Moreover, *MAP3K20*, a pro-apoptotic gene, and *TNFRSF10D*, involved in the death receptor signalling pathway, were also upregulated (Fig. 4 G). We performed GSEA pathway analysis to further characterize the differences between these two clusters, revealing the significant enrichment of the IFN-γ signalling and IFN α/β signalling pathways in NC1 compared with NC2 (Fig. 4 H).

### B cell lymphopoiesis was arrested at the pro-B cells level

**Figure 5.**
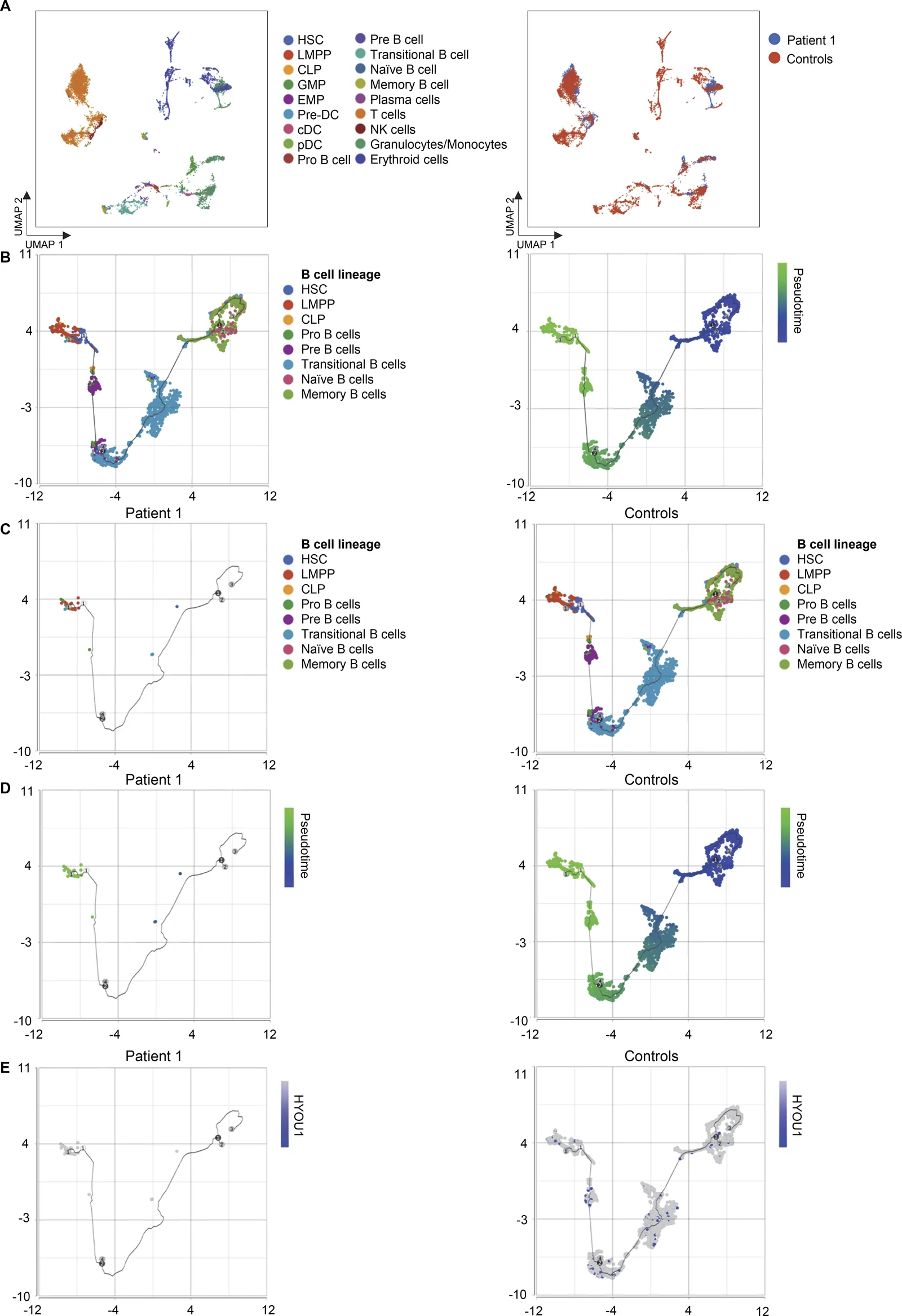
**scRNA-seq analyses of bone marrow cells. (A)** UMAP plot showing single-cell transcriptome of bone marrow cells in patient 1 and controls (left, colors correspond to cell type; right, colors correspond to samples). **(B)** Trajectory analysis of the B cell lineage of patient 1 and controls (left); pseudotime trajectory denoting the differentiation of the B cell lineage (right). **(C)** The B cell lineage of patient 1 (left) and the controls (right), according to annotation. LMPP, lympho-myeloid primed progenitors; CLP, common lymphoid progenitors. **(D)** The B cell lineage of patient 1 (left) and the controls (right) according to pseudotime analysis. **(E)** Expression of *HYOU1* in the B cell lineage of patient 1 and controls.

**Figure S5.**
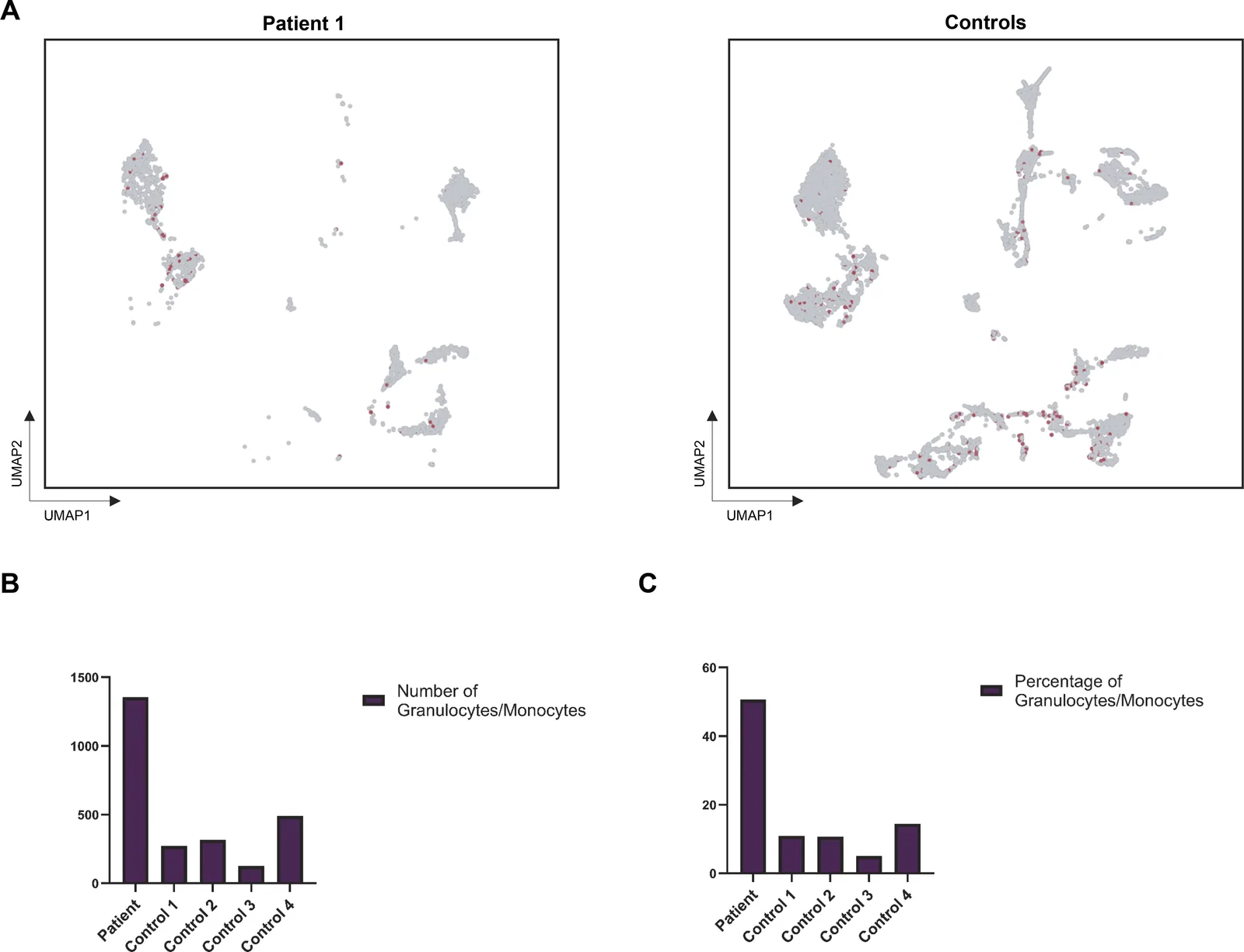
***HYOU1* expression and granulocyte/monocyte distribution in bone marrow scRNA-seq. (A)** UMAP plot showing the normalized counts for patient 1 (left) and the controls (right) in bone marrow. Red represents cells expressing *HYOU1*. **(B)** Bar graph representing the number of cells from scRNA-seq classified as granulocytes and monocytes in the bone marrow of different samples. **(C)** Bar graph representing the percentage of granulocytes and monocytes in each sample in scRNA-seq data.

In the scRNA-seq analysis of the bone marrow of patient 1, after quality control and normalization, 5,157 cells were obtained in total (*n* = 2,673 in the patient 1 and *n* = 2,484 in an age-matched control of European origin). We added three more controls from the publicly available data published by Oetjen et al., 2018 (15) (number of cells after quality control and normalization: control C2 = 2,496, control W = 3,396, and control B = 2,945). We performed PCA and used 20 PCs for further analyses. We used Harmony to correct batch effects arising from sequencing run and sex. Graph-based clustering revealed 13 clusters of cells in bone marrow. We used Azimuth (16) to map clusters to cell types guided by canonical markers; Fig. 5 A presents the UMAP with the resulting labels. *HYOU1* was not predominantly expressed by any distinct cluster (Fig. S5 A). There were more granulocyte and monocyte populations in patient 1 than in the controls (patient 1 = 1,354, 4 controls = 1,203; Fig. S5, B and C).

Trajectory analysis of the B cell lineage showed a contrast between patient 1 and controls (Fig. 5, B, C, and D). While the controls’ cells progressed continuously from hematopoietic stem cells (HSC) to naïve B cells, the patient’s failed to progress beyond the pro-B cell stage, and the trajectory stopped. The patient had no naïve or memory B cells, and the percentage of pre-B cells and transitional B cells was considerably reduced in the patient (0.0 and 0.1%, respectively) compared with the controls (1.6 and 8.5%, respectively) (Fig. 5 C). Altogether, these findings strongly suggest a maturation arrest at the pro-B cell stage to pre-B cell stage.

## Discussion

In this study, we report two new patients with immunodeficiency harboring novel variants in *HYOU1*, and we capitalize on a single patient who presented with chronic diarrhea, FTT, and episodes of hypoglycemia, to perform an extended multi-omics dissection of the disease pathophysiology. All previous case reports of patients with a variant in *HYOU1* showed recurrent infections accompanied by neutropenia (6, 8, 9) (Table 3). The patient reported by Jafari Khamirani et al. died 1 mo after showing the first symptoms (respiratory tract infection) at 3 mo of age, unlike the other 3 patients. Similar to our patient, Haapaniemi et al. also reported SGA, FTT, and episodes of stress-induced hypoglycemia. All patients except patient 1 reported in the present study had experienced respiratory infections. When considering the three published cases and the present two, anemia and skeletal anomalies were reported in three out of five patients, including our patient 1. In addition, four of the five patients had reduced numbers of B cells, including the present cases. Moreover, 2 of the patients reported before (6, 9) and patient 2 reported in this study carried compound heterozygous mutations in multiple domains of *HYOU1*. In total, neutropenia and B cell deficiency leading to recurrent infections are the prominent attributes of this phenotype, and the differences in the phenotypic spectrum could be due to patient-specific variants, their protein domain localization, or caused by somatic mutations in modifier genes.

**Table 3.** Comparison of genetic and clinical data of previously reported cases with *HYOU1* variants and patients reported in this study

​	Haapaniemi et al. (6)	Jafari Khamirani et al. (8)	Arab et al. (9)	Patient 1	Patient 2
Ethnicity	Finnish	Iranian	Iranian	Iranian	American
Age	45 years	3 mo	4 years	9 years	32 years
Sex	Female	Female	Female	Male	Male
Zygosity	Compound heterozygous	Homozygous	Compound heterozygous	Homozygous	Compound heterozygous
Variants^a^	p.Ala419Pro	p.Arg486Cys	p.Leu23Phe	p.Pro444His	p.Arg262Gln p.Pro757_Glu758insAla
	p.Tyr231His		p.Arg915Gln		
Small for gestational age	+	−	−	+	−
FTT	+	−	−	+	−
Recurrent respiratory tract infection	+	+	+	−	+
Recurrent diarrhea	+	+	−	+	+
Neurodevelopmental delay/regression	−	+	−	−	−
Herpes infection	+	−	−	−	−
Pectus carinatum	+	+	−	−	−
Facial dysmorphism	+	−	−	+	−
Hypoglycemia	+	−	−	+	−
Neutropenia	+	+	+	+	−
Reduced B cells	+	+	−	+	+
		(Pancytopenia)			
Anemia	+	+	−	+	−
Thrombocytopenia	+	+	−	+	−

a Based on NCBI Reference Sequence NP_001124463.1.

The UPR plays a fundamental and critical role in immune cell development and function that extends beyond the classical role of managing misfolded protein load. The three ER-resident sensors (IRE1α, PERK, and ATF6α) coordinate distinct transcriptional and translational outputs that are indispensable for the development and function of multiple immune cell lineages (3). Among these, the IRE1α/XBP1 axis is the most extensively characterized in immune contexts, as XBP1 is required for plasma cell differentiation and immunoglobulin secretion, for the survival of plasmacytoid and CD8α^+^ dendritic cells, and for early B lymphopoiesis at the pro-B cell stage (3). Beyond lymphoid lineages, translational control mediated by PERK-dependent eIF2α phosphorylation is critical for HSC integrity under stress, and its disruption impairs hematopoietic reconstitution capacity (3). In addition, UPR activation synergizes with innate immune signalling, as IRE1α/XBP1 directly augments type I IFN production in response to TLR ligation, and loss of this axis attenuates IFN-β induction (3). These observations collectively suggest that intact UPR signalling is a prerequisite for normal hematopoiesis, innate immune sensing, and lymphocyte maturation. In our functional assessment of patient 1–derived HDFs, we observed that when HDFs were stressed using tunicamycin, *HYOU1* transcripts were less induced in the patient, in addition to the negative enrichment of response to the ER stress pathway, signifying the inability of *HYOU1*-mutated cells to cope with stress, physiological or induced, and suggesting that *HYOU1* is involved in a self-feedback loop. These findings were independently confirmed by RT-qPCR, which demonstrated significantly reduced fold induction of five key UPR target genes (*HYOU1*, *HSPA5*, *DNAJB9*, *HERPUD1*, and *DDIT3*) in the patient following tunicamycin treatment, and by western blot showing reduced HERPUD1 protein induction. While dysregulation of *HYOU1* is already linked to ER stress–related diseases (2), we demonstrated that this variant, which leads to the absence of the HYOU1 protein, is associated with UPR activation and apoptotic pathways.

Of note, the Pro444His variant was associated with markedly reduced HYOU1 protein abundance despite preserved transcript levels, suggesting that the pathogenic effect operates at the posttranscriptional or posttranslational level rather than through impaired transcription. Several mechanisms may account for this discrepancy. The substitution of a conserved proline residue within the ATPase domain may destabilize the folded protein, rendering it a substrate for ER-associated degradation via the proteasomal pathway, resulting in reduced stable protein abundance. Alternatively, as HYOU1 is an ER-resident glycoprotein, the Pro444His substitution may interfere with posttranslational modifications required for proper folding and stability, indirectly promoting protein degradation. Reduced solubility due to mutant protein aggregation represents an additional possibility. Distinguishing between these mechanisms would require dedicated experiments including proteasome inhibition assays, solubility fractionation, and glycosylation profiling of the mutant protein, and represents a priority for future mechanistic studies. Consistent with this interpretation, western blot analysis of tunicamycin-treated HDFs showed that HYOU1 fold induction was comparable between the patient and controls, whereas the absolute induced protein level remained far below that of controls, supporting a defect in protein stability rather than in stress-responsive transcriptional induction.

A key limitation of this study is the limited experimental characterization of patient 2, due to the unavailability of biological material from this patient for research purposes. This precludes functional assessment and in-depth immunophenotyping comparable to that of patient 1. Hence, mechanistic inferences regarding HYOU1 dysfunction in patient 2 rely primarily on clinical and genetic findings. Moreover, and as mentioned above, patient 2 did not report any overt metabolic disorder and specifically any hypoglycemic episodes, especially as an adult. Further studies incorporating functional analyses will be required to confirm these observations.

While our RNA-seq experiment in assessing ER stress response in HDFs of patient 1 demonstrates a markedly impaired UPR response, it does not formally establish causality between the HYOU1 variants and the transcriptional phenotypes. Two complementary experiments would strengthen this link. First, a genetic recapitulation model, such as introduction of patient variants into control cells to confirm that variants alone are sufficient to impair the UPR. Second, a genetic rescue experiment, in which re-expression of wild-type HYOU1 in patients’ HDFs restores the ER stress response to that of the controls level. Both represent priorities for future studies, and their absence is acknowledged as a limitation of the current work. Nevertheless, the convergence of RNA-seq, RT-qPCR, and western blot data, each derived from independent biological replicates and obtained using distinct methodologies, strengthens the inference that the observed ER stress phenotype is a consequence of the HYOU1 variant rather than an artefact of any single experimental platform.

UPR activation and XBP1 activation are pivotal to plasma cell differentiation and immunoglobulin secretion (17), as well as early lymphopoiesis at the pro-B cell stage (18). Disruption of this pathway leads to defective plasma cell development and reduced antibody production, underscoring the dependence of B cell lineage progression on ER proteostasis. Owing to extensive protein synthesis and high levels of antibody production in plasma cells, ER expansion takes place prior to immunoglobulin synthesis during plasma cell differentiation. Plasma cell differentiation is known to be XBP1-dependent, and defects in XBP1 or UPR lead to a decrease in antibody production, while XBP1 deficiency results in the absence of plasma cells (2). Other studies have shown that UPR is activated in pro-B cells and pre-B cells (17, 18), which is consistent with our observation of the suggested stage at which the arrest of B cell differentiation occurs in the patient. UPR and XBP1 activation are pivotal to plasma cell differentiation and immunoglobulin secretion (17), as well as early lymphopoiesis in the stage of pro-B cells (18) or even neutropenia (19). Although the exact position of HYOU1 within the UPR signalling cascade is not precisely known, HYOU1 is known to play a cytoprotective and anti-apoptotic role in response to ER stress (2). Our findings suggest that the absence of HYOU1 is associated with differentiation arrest before the pro-B cell stage, leading to a markedly reduced number of B cells in the bone marrow and blood. In this context, the reduced induction of UPR-related genes observed in the patient following tunicamycin treatment suggests that HYOU1 deficiency may disrupt this critical stress–adaptation pathway, which could in part contribute to the observed defects in B cell development and function. The reduction in B cell marker expression was further confirmed at the transcript level by RT-qPCR on PBMCs, which showed significantly decreased *CD79A* and *MS4A1* in the patient compared with heterozygous family controls, providing orthogonal validation of the multi-omics findings.

Moreover, we showed that the neutrophils were hypogranulated in the BM of patient 1 prior to G-CSF administration, and hypogranulation of neutrophils continued after receiving G-CSF. Although the expression of certain genes in neutrophils was altered, according to the patterns previously described (20, 21), IFN-related genes were downregulated in response to G-CSF in our patient, unlike the healthy individuals receiving G-CSF treatment reported in these studies. These data show that not all changes in neutrophil transcripts are caused by G-CSF. Altogether, these findings indicate that the hypogranulation and lack of response to IFNs are inherent characteristics of the patient’s neutrophils and that the *HYOU1* variant rather impacts neutrophils’ morphology, granule content, and function than numbers. Previous studies support the synergistic influence of UPR activation upon IFN-β synthesis (22), suggesting that impairment in UPR activation and XBP1 splicing plays a role in the downregulation of response to type I IFN in the neutrophils of the patient. RT-qPCR validation on PBMCs further showed elevated expression of *MMP9* and *TNFRSF1B* in the patient, consistent with the transcriptomic findings of upregulated secretory granule and TNF signalling pathways.

Previous studies also demonstrated that the UPR is a pivotal modulator of skeletal metabolism and homeostasis and is crucial for managing physiological stress caused by production of extracellular matrix proteins (7). The UPR is also involved in the differentiation of osteoblasts, chondrocytes, and osteoclasts. Defects in the UPR were shown to be associated with multiple skeletal disorders such as osteoporosis and congenital skeletal disorders (23). Upregulation of the osteoclast differentiation pathway in the RNA-seq of bone marrow of patient 1 may suggest that FTT and SGA in the patient could be due to the role of the UPR in the differentiation of osteoclasts.

In clinical practice, several IEIs can cause neutropenia alongside a reduction in B lymphocytes and should be considered in the differential diagnosis of HYOU1 deficiency. The combination of neutropenia and B cell deficiency raises the suspicion of X-linked agammaglobulinemia, certain forms of SCID associated with RAG mutations, ADA deficiency, and complex IEIs affecting multiple myeloid and lymphoid lineages, such as mutations in GATA2, AK2, and IKZF1 (24, 25, 26, 27, 28, 29, 30). However, the distinguishing feature of HYOU1 deficiency is the concurrent impairment of both myeloid maturation and lymphoid development in the context of ER stress dysfunction, with neutrophil hypogranulation and a maturation arrest at the promyelocyte stage alongside a profound reduction in B cells and dendritic cells. This combination of findings, particularly when associated with hypoglycemia and evidence of impaired UPR activation, should prompt consideration of HYOU1 deficiency and guide targeted genetic investigation.

Taken together, we further describe the phenotypic spectrum associated with novel recessive *HYOU1* variants in 2 unrelated patients; we demonstrate that the HYOU1 Pro444His affects neutrophil function and morphology, and show that B cell development is most probably arrested at the pro-B cells stage. These features may contribute to impairment in innate and adaptive immunity in patients with variants in *HYOU1*.

## Materials and methods

### Subjects, study approval, and sampling

Family 1 was of Iranian origin. The parents and brother of the proband were healthy. All subjects (or their legal guardians) gave written informed consent to participate in this study, which was conducted according to the principles of the Helsinki Declaration and approved by the institutional review board of Tehran University of Medical Sciences (Tehran, Iran). Additionally, an unrelated patient (patient 2) from the United States was included in the article for comparative clinical reporting. No biological sample was obtained from this patient, and no experimental procedure was performed. This patient gave written informed consent for publication of his clinical data.

Bone marrow and skin biopsies were obtained from patient 1. Blood sampling was further carried out for patient 1 and his family. In total, sampling for the proband’s blood was done four times over the course of 2 years (which were used as biological replicates). The timing of G-CSF administration relative to each sample collection date, alongside the analyses performed on each shipment, is summarized in Table S1. PBMCs were isolated within 24 h of sampling using standard Ficoll-Paque density gradient centrifugation.

### Exome sequencing

Genomic DNA was isolated from the peripheral blood of patient 1 (II.1), his parents (I.1, I.2), and his healthy brother (II.2) using standard protocols. Exome sequencing was performed on a NextSeq500 sequencer (Illumina) as described previously (31). The following filtering steps were applied for variant identification in the proband, i.e., selecting only (1) variants with a depth of coverage of more than or equal to 10 reads; (2) protein-altering variants (excluding noncoding or synonymous variants); (3) variants with allele frequencies <0.001 in the 1000 Genomes Project, gnomAD, and an internal database including >2,000 exomes; and (4) variants homozygous in the patient, heterozygous in parents, heterozygous, or wildtype in the healthy brother. Raw exome files are available at the National Center for Biotechnology Information (accession number BioProject: PRJNA1214550). Patient 2’s exome was done in a clinical setting.

### Sanger sequencing

The three candidate variants were confirmed by Sanger sequencing as described earlier (31) and using the following primers for both, amplification and sequencing: 5′-GCG​GGA​CCC​TTT​TCT​GTT​GTC​ATC-3′ (p.Pro444His, forward); 5′-GGA​TCT​CCT​GAG​GGC​TAT​GTC​ACA​G-3′ (p.Pro444His, reverse); 5′-GCG​GGA​CCC​TTT​TCT​GTT​GTC​ATC-3′ (p.Arg262Gln, forward); 5′-GGA​TCT​CCT​GAG​GGC​TAT​GTC​ACA​G-3′ (p.Arg262Gln, reverse); 5′-GAT​AAG​CTG​GCT​CAG​TCG​GTG​C-3′ (p.Pro757_Glu758insAla, forward); 5′-CAT​TGA​TGA​CTT​TCT​CTA​ACG​TTG​TCA​TCT​CC-3′ (p.Pro757_Glu758insAla, reverse).

### Transcriptome and proteome analyses

Bulk RNA sequencing was performed on bone marrow, HDF, and PBMCs of patient 1 and compared with the corresponding sample types from healthy controls. Total RNA was extracted using the RNeasy Mini Kit (74106; Qiagen). Total RNA-seq library preparation and sequencing were performed as previously described (31). For each sample, quality control was carried out and assessed with the NGS Core Tools FastQC (https://www.bioinformatics.babraham.ac.uk/projects/fastqc/). The RNA-seq analysis was carried out using the bcbio-nextgen framework, an open-source toolkit. RNA-seq reads were quantified against the *Homo sapiens* hg19 (GRCh37) genome using the pseudoaligner Salmon (32). Differential expression analysis was conducted with DESeq2 (33) in the R programming environment. Up- and downregulated genes were selected based on the adjusted P value (<0.05) and the log2-fold-change (≥1). Raw RNA-seq data were deposited in the EMBL-EBI ArrayExpress archive under the accession number E-MTAB-15443.

Proteomics analyses were performed on HDFs and PBMCs. Protein extraction, enzymatic digestion, HPLC separation, analysis of the resulting peptides with nano-liquid chromatography/tandem mass spectrometry, and software-based protein quantification using MaxQuant were performed following the previous protocols described (31). Up- and downregulated proteins were selected based on the adjusted P value (<0.05) and the log2-fold-change (≥1). The complete proteomics dataset was deposited into the ProteomeXchange Consortium via the PRIDE partner repository under the accession number PXD058947.

Gene Ontology and pathway analyses of differentially expressed genes and proteins were performed using the WebGestalt toolkit (34) (www.webgestalt.org). The functional databases of non-redundant biological process, nonredundant cellular process, and nonredundant molecular process were used for Gene Ontology analyses. The functional databases of KEGG, Panther, and Reactome were used for pathway analyses.

### scRNA-seq

scRNA-seq was performed on whole blood and bone marrow samples following the same quality control and sequencing procedures described above, with the following differences. 500 µl of whole blood or bone marrow were incubated twice for 10 min at room temperature (RT) with RBC lysis solution 1X (Miltenyi). Cells were then washed and centrifuged at 500 × *g* and subsequently resuspended in Dulbecco’s PBS (14190144; Gibco, Thermo Fisher Scientific) containing 2% FBS for cell counting, using Trypan Blue and Countess II FL (Invitrogen, Thermo Fisher Scientific). Libraries were prepared using the Chromium Single Cell 3′ Library and Gel Bead kit v3.1, Chip G kit, Single Index kit T set A, and the Chromium Controller according to the manufacturer’s instructions (10X Genomics). Cells were loaded at a volume to target around 5,000 cells per sample. Libraries were sequenced using an Illumina NextSeq 2000 instrument with a sequencing depth of at least 30,000 reads per cell. Raw scRNA-seq data were deposited in the EMBL-EBI ArrayExpress archive under accession number E-MTAB-14786. Raw-sequencing data were processed using the Cell Ranger analysis pipeline, v6.1.2 (35) to capture neutrophils from 3′ Single Cell Gene Expression data. Downstream analyses were performed using Partek Flow software v10.0 (https://www.partek.com/partek-flow/). The following filtering criteria were used to remove low-quality cells with (1) <500 and >15,000 genes and/or (2) <300 and >5,000 UMI counts and/or (3) >10% mitochondrial gene counts. In blood, this resulted in a total of 28,757 cells, 13,021 cells for patient 1 (merged from three independent samplings), and 15,736 cells for the controls (4 controls including patient’s healthy brother). In bone marrow, the total residual cells were 5,139 cells, 2,668 for patient 1, and 2,471 for the control. Normalization was conducted using counts per million with log2 transformation. Batch effect correction was applied using Harmony in the PCA space, followed by graph-based clustering using 20 PCs and UMAP visualization. Pseudotime trajectory analysis was performed using the Monocle 3 algorithm within Partek Flow, with the root node selected manually based on known cell annotation. Differentially expressed genes between clusters were identified using gene-specific analysis based on the limma-trend method.

### Structural modelling

The full-length human HYOU1 protein sequence (UniProt ID: q9y4l1 hyou1_human) was used for all structural modelling analyses. To identify potential structural templates, a SWISS-MODEL (36) search against the Protein Data Bank (PDB) (37) was performed. The NTD showed high-confidence alignment to the structure 7A4U (38), which was selected as the primary template for NTD modelling. Homology models were generated using SWISS-MODEL with default parameters. A hybrid full-length model was constructed by combining the 7A4U-based NTD with AlphaFold3-predicted (39) middle and CTDs. Structural alignment was performed in PyMOL v2.5 (https://pymol.org/) using the align function, with the middle domain serving as an anchor to guide the assembly of the NTD and CTD. The RMSD between the template-based NTD and the AlphaFold3-derived middle domain was calculated to evaluate fit. Regions predicted with low AlphaFold3 confidence were retained only for qualitative context. The three HYOU1 variants—p.Arg262Gln, p.Pro444His, and p.Pro757_Glu758insAla—were introduced into the hybrid structure using PyMOL’s mutagenesis tool. For each variant, hydrogen bonding patterns, steric interactions, and secondary-structure context were examined. Measurements of interatomic distances and hydrogen bond geometries were computed in PyMOL. The effects of the variants were evaluated through visual inspection of side-chain positioning, potential loss or gain of stabilizing interactions, and disruption of helical or β-strand boundary residues. All structural figures were generated in PyMOL, with consistent orientation and color schemes for clarity.

### HDFs isolation, culture, and stress induction

HDF isolation from patient 1 was performed within 3 days of the skin biopsy. The biopsy was first transferred to a sterile petri dish. Epidermis and hypodermis were removed and discarded. Dermis was cut into small pieces and placed in 25 cm^2^ flasks. Culture media (Dulbecco’s modified Eagle medium; 10566016, Gibco, Thermo Fisher Scientific) (DMEM) supplemented with 10% inactivated FBS, 40 U/ml penicillin, and 50 mg/ml streptomycin was added to cover the surface after 15 min. The media was changed after 24 h. The cells were passed at 80% confluency using trypsinization. Control HDFs were purchased from PromoCell.

Half a million HDFs of patient 1 and three controls at passage five were each transferred to 100-mm dishes and treated with DMEM (10566016; Gibco, Thermo Fisher Scientific) supplemented with 10% FBS, 40 U/ml penicillin, and 50 mg/ml streptomycin, 24 h prior to stress induction. ER stress was induced using 0.5 µg/ml tunicamycin (3516; TOCRIS) dissolved in DMSO (D4540; Sigma-Aldrich), and the same volume of DMSO was used for mock-treated samples. The cells were then harvested 6 h after treatment using TrypLE Express Enzyme (12605010; Gibco, Thermo Fisher Scientific). This experiment was done in three biological replicates.

### Western blotting

Frozen cell pellets of 10^6^ HDFs were thawed on ice and treated with lysis buffer (50 mM Tris-HCl, pH 8, 150 mM NaCl, 1% NP-40, 0.1% SDS, 0.1% Na deoxycholate, 1 mM dithiothreitol, 1 mM EDTA, and protease inhibitor cocktail; Roche). Cells were incubated for 20 min on ice; 20 µg of each sample was added to 5 μl of 5× loading buffer. Samples were heated for 15 min at 95°C, loaded on a 4–20% pre-cast gradient gel (NB10420; NuSep), and subjected to electrophoresis at 150 V for 70 min. Protein transfer was performed using TransBlot Turbo Transfer System (Bio-Rad Laboratories) following the manufacturer’s instructions. Membranes were blocked for 1 h with Tris-buffered saline, Tween 20 (0.05%), and nonfat milk (5%) and were incubated at 4°C overnight with anti-HYOU1 antibody (1:5,000 dilution, ab134944; Abcam; RRID:AB_2858190) for baseline protein quantification, or with anti-HYOU1 (PA5-27655; Thermo Fisher Scientific; RRID:AB_2545131) for ER stress validation experiments. For HERPUD1 protein detection in ER stress induction experiments in HDFs, membranes were probed with anti-HERPUD1 antibody (ab150424; Abcam; RRID:AB_2857374). Anti-GAPDH antibody (1:10,000, MAB374; Sigma-Aldrich; RRID:AB_2107445) was used for the detection of the housekeeping protein. Membranes were then incubated with secondary antibodies (Goat Anti-Rabbit [H+L]-HRP conjugates; 1721019; Bio-Rad Laboratories; RRID:AB_11125143, and Goat Anti-Mouse IgG [H+L]-HRP conjugates; 1706516;, Bio-Rad Laboratories; RRID:AB_2921252) at a dilution of 1:10,000 for 1 h. The ChemiDoc XRS+ system (Bio-Rad Laboratories) and Clarity Western ECL (1705061; Bio-Rad Laboratories) were used for signal detection. Western blot quantification was performed using ImageJ (40).

### Quantitative real-time PCR

Total RNA was extracted from frozen dry pellets of 10^6^ HDFs with the RNeasy Mini Kit (74106; Qiagen) and later reverse transcribed using Maxima H Minus cDNA Synthesis Master Mix (M1662; Thermo Fisher Scientific). Quantitative real-time PCR was run on a QuantStudio 3 (Thermo Fisher Scientific) using PowerTrack SYBR Green Master Mix (A46109; Thermo Fisher Scientific). The following PCR conditions were used: 10 min at 96°C, followed by 40 cycles of 95°C for 10 s and 60°C for 30 s.

For HDF analyses assessing baseline HYOU1 expression, ACTB (β-actin) was used as a housekeeping gene with the following primers: *HYOU1*, 5′-GCA​CCA​TTG​TGA​CCT​ACC​AGA-3′ (forward) and 5′-CCA​GGG​TAC​GGT​CAA​ATC​C-3' (reverse); *ACTB*, 5′-CAC​CAT​TGG​CAA​TGA​GCG​GTT​C-3′ (forward) and 5′-AGG​TCT​TTG​CGG​ATG​TCC​ACG​T-3' (reverse). For HDF ER stress validation and PBMC analyses, *GAPDH* and *HPRT1* were used as housekeeping genes, and their geometric mean was used for normalization. PrimeTime qPCR Primer Assays (Integrated DNA Technologies) were used for target amplification: *HYOU1* (Hs.PT.58.39030385), *HSPA5*/BiP/GRP78 (Hs.PT.58.22715160), *DNAJB9*/ERdj4 (Hs.PT.58.19679), *DDIT3*/CHOP (Hs.PT.58.39204289.g), *HERPUD1* (Hs.PT.58.21409911), *MS4A1* (Hs.PT.56a.24784282), *CD79A* (Hs.PT.58.21142675), *CD19* (Hs.PT.56a.20036907.g), *CD14* (Hs.PT.56a.3118607.g), *MMP9* (Hs.PT.58.22814824.g), *LCN2* (Hs.PT.58.27617685), IL1B (Hs.PT.58.20988729), and *TNFRSF1B* (Hs.PT.58.1424210). *GAPDH* primers were as follows: 5′-GGT​GAA​GGT​CGG​AGT​CAA​CGG​A-3′ (forward) and 5′-GAG​GGA​TCT​CGC​TCC​TGG​AAG​A-3' (reverse). Relative gene expression levels were calculated using the ΔΔCt method.

### Flow cytometry

Cells from 250 μl of whole blood were washed twice with 2 ml of 1X PBS, spun at 350 × *g* for 5 min at RT. The samples were then stained with 1 ml of 1:1,000 diluted Live/Dead Blue for 20 min at RT. Samples were then washed with 2 ml of FACS buffer (1X PBS, 0.5% FBS, 2 mM EDTA, and 10 mM Hepes) and collected by centrifugation at 300 × *g* for 5 min at RT. 5 μl of Human TruStain FcX and 5 μl of True-Stain Monocyte Blocker were added to each sample, vortexed, and incubated for 5 min. The mix of 40 antibodies was added in 3 batches (Table S4). After staining for 30 min on ice, 3 ml of red blood cell lysis buffer (Versalyse, Beckman Coulter Life Sciences) was added to each sample. After 10 min of incubation at RT, the cells were immediately centrifuged at 300 × *g* for 5 min at 4°C. Samples were washed twice with 2 ml of FACS buffer, centrifuged, and resuspended in 300 μl of FACS buffer. The resuspended material was then analyzed using a Cytek Aurora 5L spectral flow cytometer (Cytek Biosciences). A total of 300,000 CD45^+^ live cells were acquired. Whole-blood samples were analyzed using the OMIQ software from Dotmatics (www.omiq.ai, www.dotmatics.com). Cells were pre-gated as follows: singlets, live, time, and CD45^+^. Computational analysis was carried out on FlowSOM (41) to delineate the total number of clusters generated. Cell cluster annotations were performed using OMIQ. All frequencies of clusters in the bar plots delineated were calculated by dividing each cluster by all clusters.

### Statistics

Graphs were generated using GraphPad Prism v10.2.3 for Windows (GraphPad Software, www.graphpad.com), R packages ggplot and ggrepel. A *t* test was used for statistical comparison. Statistical comparisons between patient and control groups were performed using unpaired two-tailed Student’s *t* test unless otherwise stated. Actual P values are reported in each figure legend; a P value <0.05 was considered statistically significant. Data are presented as mean ± SD unless otherwise indicated. The number of patient and control samples included in each experimental analysis is summarized in Table S5.

### Online supplemental material

Supplemental material includes five supplemental figures (Figs. S1, S2, S3, S4, and S5) and five supplemental tables (Tables S1, S2, S3, S4, and S5). Fig. S1 shows structural modelling of the HYOU1 protein. Fig. S2 shows RT-qPCR validation of UPR gene induction in tunicamycin-treated HDFs as well as RT-qPCR validation of B cell and innate immune markers in PBMCs. Fig. S3 shows western blot analysis of HERPUD1 and HYOU1 protein induction following ER stress. Fig. S4 shows marker gene expression used for single-cell cluster annotation in peripheral blood. Fig. S5 shows HYOU1 expression and cell composition in bone marrow single-cell data. Table S1 shows additional laboratory results for both patients. Table S2 lists the remaining candidate variants and their annotations. Table S3 summarizes the timing of G-CSF administration relative to each sample collection date and the analyses performed on each shipment. Table S4 lists the antibodies used for spectral flow cytometry. Table S5 summarizes the number of patient and control samples per experimental analysis. Raw RNA-seq data were deposited in the EMBL-EBI ArrayExpress archive (E-MTAB-15443), raw scRNA-seq data in the EMBL-EBI ArrayExpress archive (E-MTAB-14786), raw exome sequencing data in the NCBI BioProject (PRJNA1214550), and proteomics data in the ProteomeXchange Consortium via PRIDE (PXD058947).

## Supplementary Material

Table S1 shows extensive clinical laboratory data for both patients.

Table S2 shows annotation, predicted impact, and population frequencies of candidate variants after segregation analysis in patient 1.

Table S3 shows sampling and shipments for patient 1 with respect to G-CSF administration.

Table S4 shows antibodies used in immunophenotyping.

Table S5 shows sample size for patients and controls per tissue and experiment.

SourceData F1 is the source file for Fig. 1.

SourceData FS3 is the source file for Fig. S3.

## Data Availability

All data underlying this study are available. Raw bulk and scRNA-seq data have been deposited in the EMBL-EBI ArrayExpress archive under accession numbers E-MTAB-15443 and E-MTAB-14786, respectively. Raw exome sequencing data are available at the National Center for Biotechnology Information under BioProject accession PRJNA1214550. The complete proteomics dataset has been deposited into the ProteomeXchange Consortium via the PRIDE partner repository under accession number PXD058947. The ClinVar IDs of the variants reported here are SCV007596332 for p.Pro444His (patient 1), SCV007596333 for p.Arg262Gln (variant#1 of patient 2), and SCV007596399 for p.Pro757_Glu758insAla (variant#2 of patient 2). All other data supporting the findings of this study are available within the article and its supplemental material.
