# Supplementary material for: ER proteostasis failure in HYOU1 deficiency alters B cells, neutrophils, and interferon signalling
Source: J Hum Immun. 2026 Sep 7;2(6):e20250234. doi: 10.70962/jhi.20250234 (PMC13549398; doi:10.70962/jhi.20250234)
Supplement: Supplementary file 1 [file jhi_20250234_sourcedataf1.pdf]

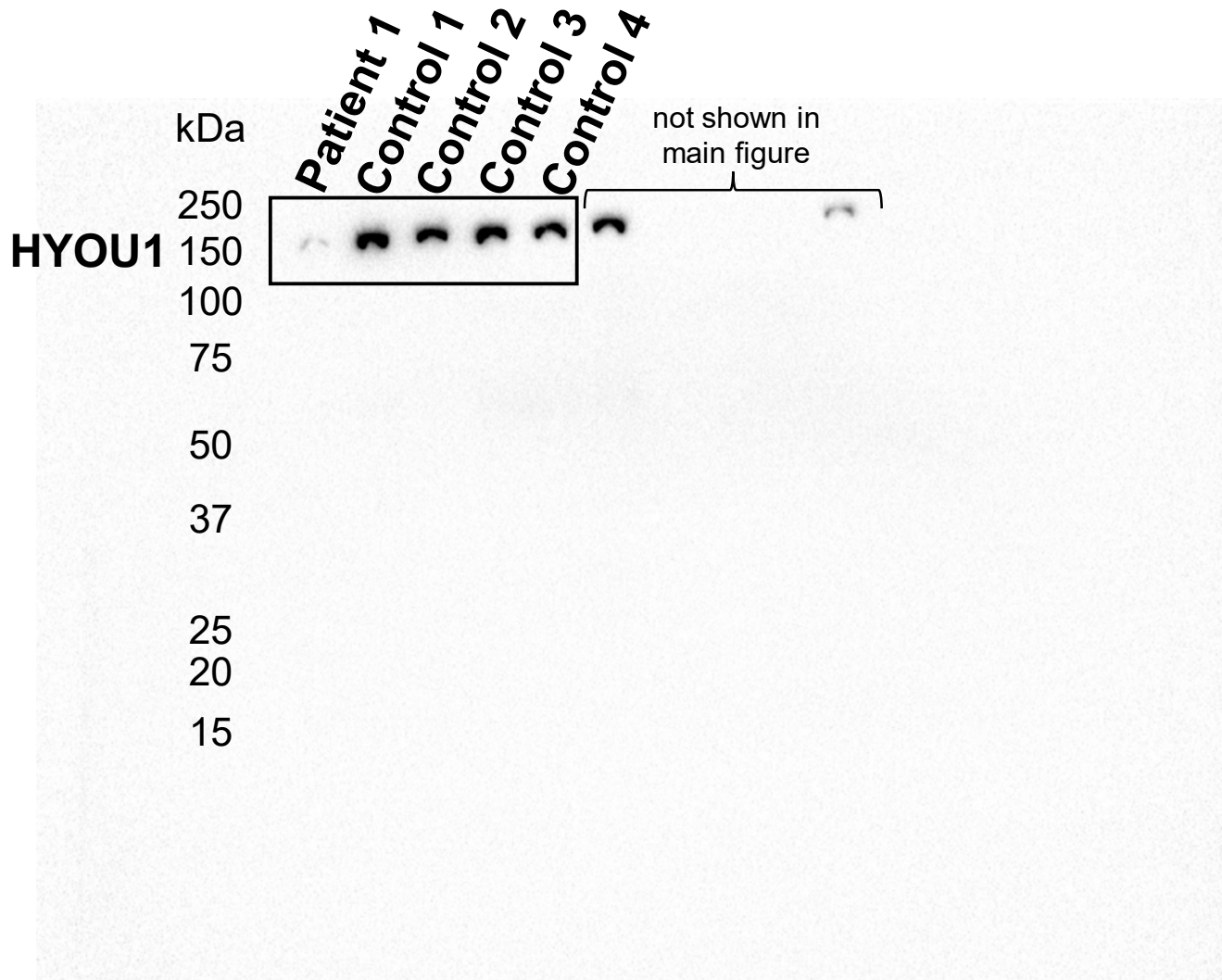
kDa
Patient 1
Control 1
Control 2
Control 3
Control 4
not shown in
main figure
250
HYOU1
150
100
75
50
37
25
20
15

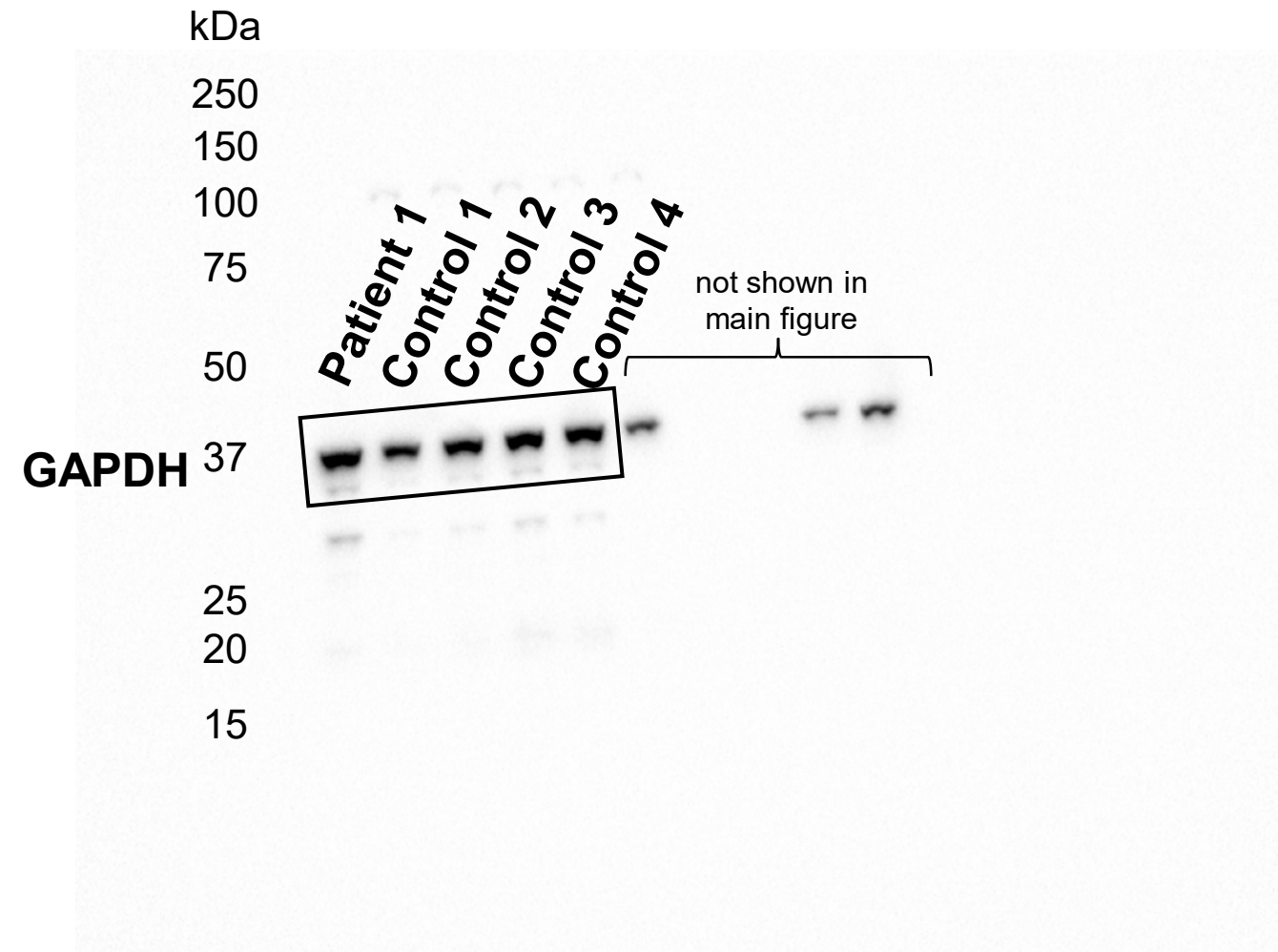
kDa
250
150
100
75
Patient 1
Control 1
Control 2
Control 3
Control 4
not shown in
main figure
50
GAPDH
37
25
20
15
