# Supplementary material for: ER proteostasis failure in HYOU1 deficiency alters B cells, neutrophils, and interferon signalling
Source: J Hum Immun. 2026 Sep 7;2(6):e20250234. doi: 10.70962/jhi.20250234 (PMC13549398; doi:10.70962/jhi.20250234)

**Figure SF3C**

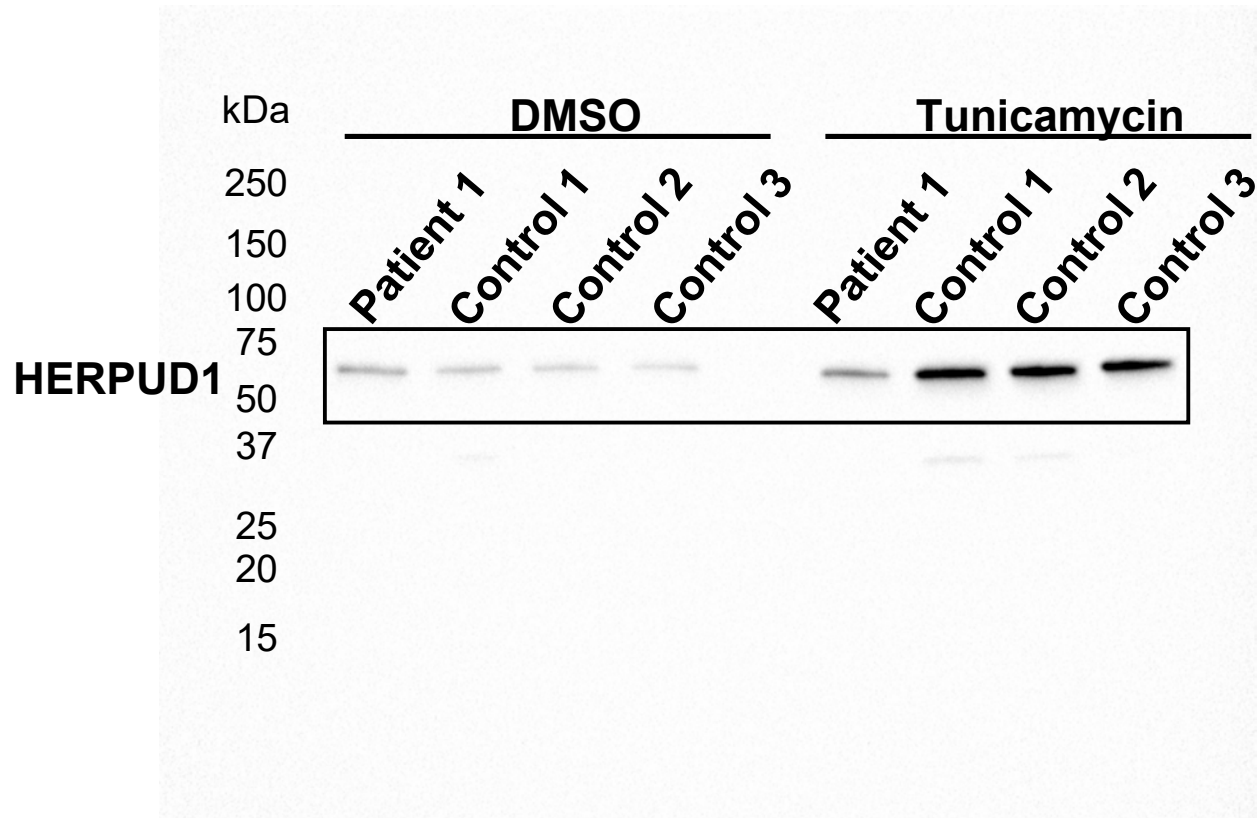


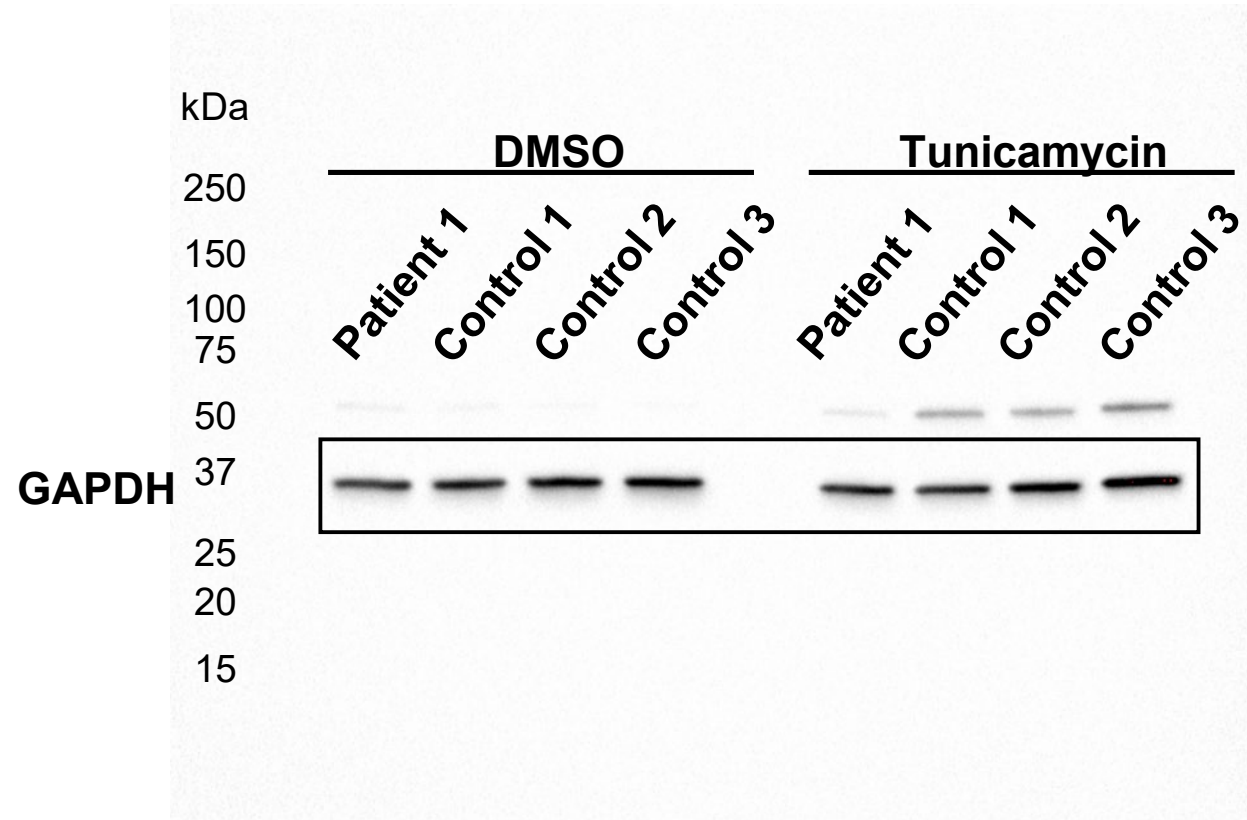


**Figure SF3D**

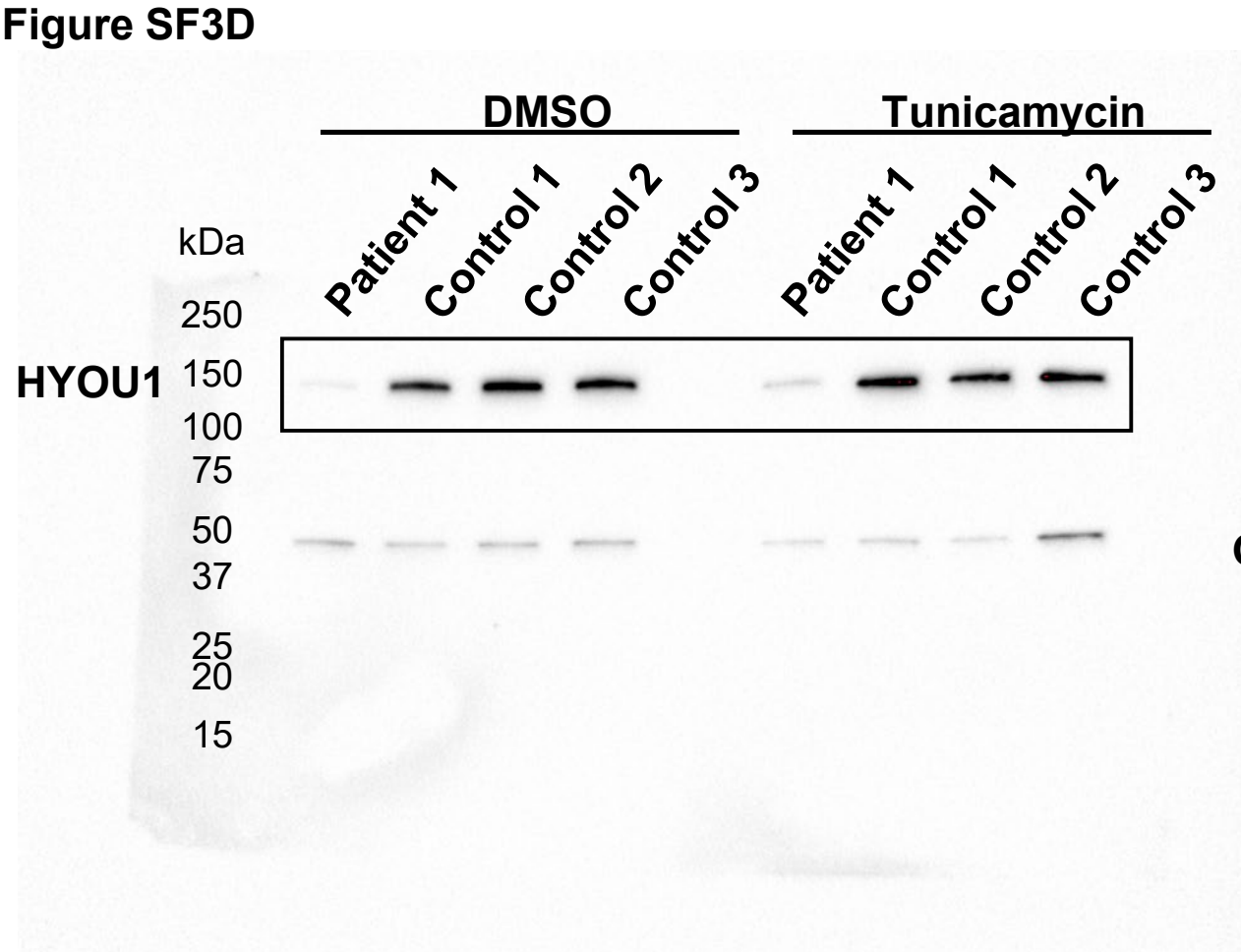


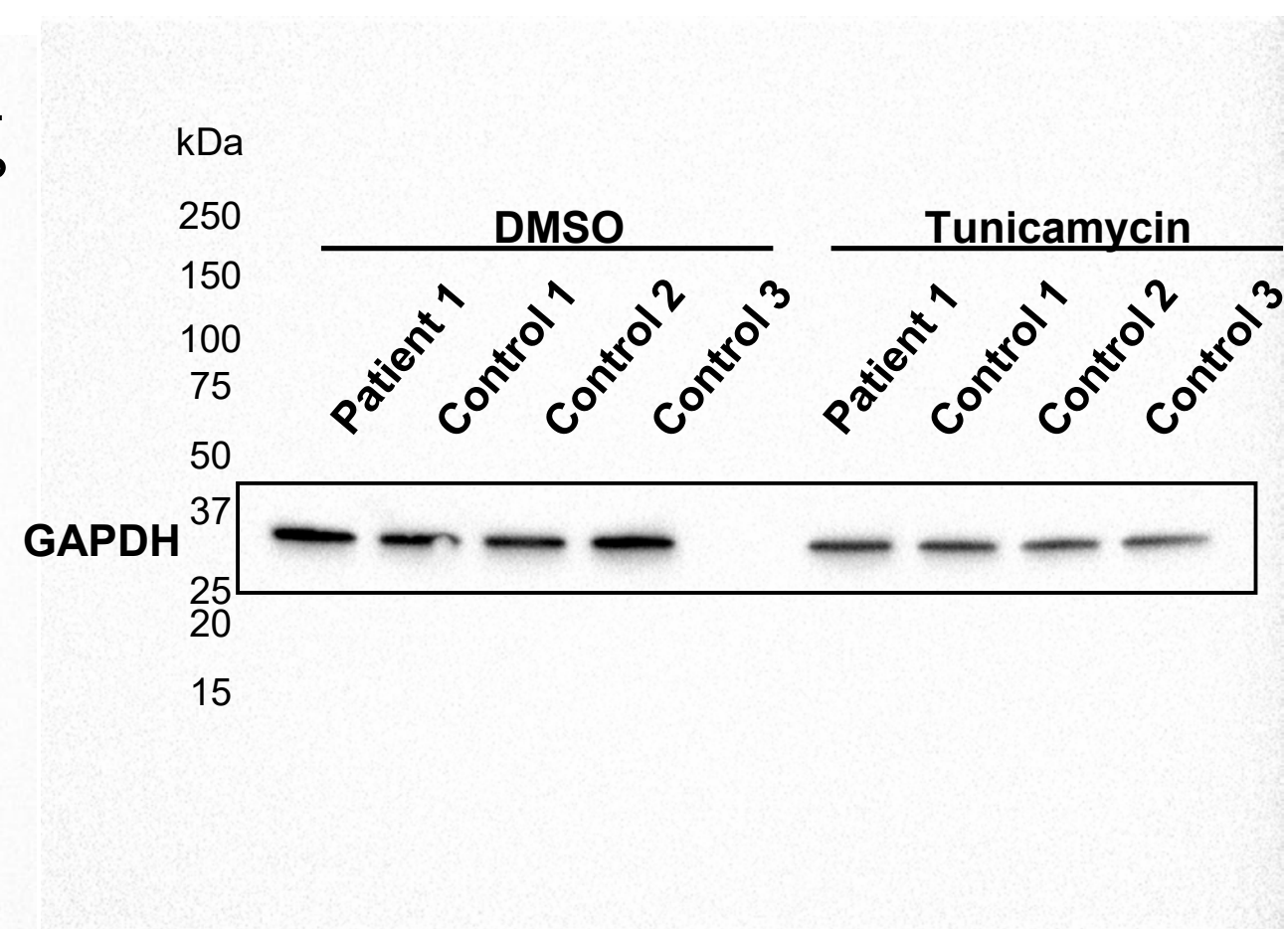

Supplement: Supplementary file 2 [file jhi_20250234_sourcedatafs3.pdf]
