# Supplementary material for: ER proteostasis failure in HYOU1 deficiency alters B cells, neutrophils, and interferon signalling
Source: J Hum Immun. 2026 Sep 7;2(6):e20250234. doi: 10.70962/jhi.20250234 (PMC13549398; doi:10.70962/jhi.20250234)
Supplement: Supplementary file 3 [file jhi_20250234_tables2.docx]

**Table S2. Annotation, predicted impact, and population frequencies of candidate variants after segregation analysis in Patient 1.**

| Variant annotation, predicted impact and population frequencies | | | | | | | |
| --- | --- | --- | --- | --- | --- | --- | --- |
| Position (hg38) | chr1:158356534 | chr2:241063661 | chr10:71687725 | chr11:6546014 | chr11:117917279 | chr11:124987209 | chr13:113233973 |
| Gene symbol | *CD1E* | *SNED1* | *CDH23* | *DNHD1* | *TMPRSS13* | *CCDC15* | *CUL4A* |
| Ref. allele | C | C | G | G | G | T | T |
| Alt. allele | G | T | A | A | A | G | C |
| dbSNP | rs756511340 | rs374311632 | rs727502923 | - | rs1480573177 | - | rs142598917 |
| Consequence | missense | missense | splice region and intron variant | missense | splice region and intron variant | missense | missense |
| HGVSc: nucleotide change | NM_001042583.3:c.941C>G | NM_001080437.3:c.2446C>T | NM_001171930.2:c.2059+6G>A | NM_144666.2:c.5075G>A | NM_001077263.3:c.452-5C>T | NM_025004.3:c.983T>G | NM_001008895.4:c.752T>C |
| HGVSp: protein change | NP_001036048.1:p.Thr314Ser | NP_001073906.1:p.Arg816Trp | - | NP_653267.2:p.Gly1692Asp | - | NP_079280.2:p.Ile328Ser | NP_001008895.1:p.Met251Thr |
| Genotype |  |  |  |  |  |  |  |
| Patient | 1/1 | 1/1 | 1/1 | 1/1 | 1/1 | 1/1 | 1/1 |
| Brother | 0/1 | 0/0 | 0/1 | 0/1 | 0/0 | 0/0 | 0/1 |
| Father | 0/1 | 0/1 | 0/1 | 0/1 | 0/1 | 0/1 | 0/1 |
| Mother | 0/1 | 0/1 | 0/1 | 0/1 | 0/1 | 0/1 | 0/1 |
| Predictive scores of pathogenicity | | | | | | | |
| SIFT (score)^a^ | Deleterious (0.04) | Tolerated (0.17) |  | Deleterious (0) |  | Deleterious (0) | Deleterious (0.03) |
| PolyPhen (score)^b^ | Benign (0.228) | Possibly damaging (0.903) |  | Probably damaging (1) |  | Probably damaging (0.996) | Benign (0.018) |
| CADD (phred score)^c^ | 10.46 | 23.6 | 7.58 | 24.3 | 9.029 | 22.5 | 22.2 |
| GERP++_RS^d^ | 2.44 | 3.52 |  | 5.56 |  | 2.62 | 4.76 |
| MutationTaster | 0.999999 | 0.999704 |  | 0.999615 |  | 0.995605 | 1 |
| phastCons20way_mammalian^e^ | 0.10243 | 0.58386 |  | 0.66667 |  | 0.49956 | 0.55488 |
| ClinVar ClinSig |  |  | Uncertain significance |  |  |  |  |
| Allele Frequencies |  |  |  |  |  |  |  |
| gnomAD combined populations | 0.00003209 | 0.00006952 | 0.00003235 |  | 0.000004142 |  |  |
| gnomAD_AFR | 0 | 0.00006674 | 0.00006582 |  | 0 |  |  |
| gnomAD_AMR | 0 | 0 | 0.00005815 |  | 0 |  |  |
| gnomAD_ASJ | 0 | 0.000403 | 0.0001001 |  | 0 |  |  |
| gnomAD_FIN | 0 | 0 | 0 |  | 0 |  |  |
| gnomAD_NFE | 0 | 0.0001086 | 0.00002683 |  | 0 |  |  |
| gnomAD_number of homozygous | 0 | 0 | 0 | 0 |  |  |  |
| In house allele count^f^ | 0 | 0 | 0 | 0 | 0 | 0 | 0 |
| In house allele frequency | 0 | 0 | 0 | 0 | 0 | 0 | 0 |
| In house number of homozygous | 0 | 0 | 0 | 0 | 0 | 0 | 0 |
| In house number of heterozygous | 1 | 0 | 0 | 0 | 0 | 0 | 0 |

a Function prediction tool based on protein sequence conservation among homologs. Variants with scores between 0 and 0.05 are considered deleterious.

b Polyphen2_HDIV_score: variants with scores between 0.85 and 1.0 are predicted to be damaging with high confidence.

c CADDv1.4 scores range from 1 to 99, with a higher score indicating greater deleteriousness.

d GERP++_RS score: DNA conservation score. Scores range from 1 to 6.18. The larger the score, the more conserved the site.

e PhastCons score: probabilities of negative selection and range between 0 and 1.

f based on an in-house database of ~2000 exomes
