# Supplementary material for: ER proteostasis failure in HYOU1 deficiency alters B cells, neutrophils, and interferon signalling
Source: J Hum Immun. 2026 Sep 7;2(6):e20250234. doi: 10.70962/jhi.20250234 (PMC13549398; doi:10.70962/jhi.20250234)
Supplement: Supplementary file 4 [file jhi_20250234_tables3.docx]

**Table S3. Sampling and shipments for Patient 1 with respect notably to G-CSF administration.**

| **Shipment** | **Date of Sampling** | **G-CSF* to sampling interval** | **Bulk RNAseq**  **BM** | **scRNAseq**  **BM** | **Bulk RNAseq PBMC** | **scRNAseq Blood** | **Proteomics**  **PBMC** | **Flow**  **Cytometry** |
| --- | --- | --- | --- | --- | --- | --- | --- | --- |
| **1** | 22/11/2020 | 8 days prior | ✓ | ✓ | ✓ | ✓ | ✓ |  |
| **2** | 28/06/2021 | Day of sampling |  |  | ✓ | ✓ | ✓ |  |
| **3** | 23/02/2022 | 9 days prior |  |  | ✓ | ✓ |  | ✓ |
| **4** | 21/04/2022 | 1 day prior |  |  |  |  | ✓ | ✓ |

* BM, bone marrow; PBMC, peripheral blood mononuclear cells; scRNAseq, single-cell RNA sequencing. G-CSF (pegylated filgrastim, 100 µg/kg) was administered every 14 days. The interval between the last G-CSF dose and sample collection is indicated for each shipment. HDF experiments are not included as fibroblasts were cultured in vitro for multiple passages, rendering G-CSF timing irrelevant.
