# Supplementary material for: ER proteostasis failure in HYOU1 deficiency alters B cells, neutrophils, and interferon signalling
Source: J Hum Immun. 2026 Sep 7;2(6):e20250234. doi: 10.70962/jhi.20250234 (PMC13549398; doi:10.70962/jhi.20250234)
Supplement: Supplementary file 5 [file jhi_20250234_tables4.docx]

**Table S4. Antibodies used in immunophenotyping**

| # | Antigen | Fluorochrome | Supplier | Reference | Clone |
| --- | --- | --- | --- | --- | --- |
| 1 | CD123 | Super Bright 436 | Thermo Fisher | 62-1239-42 | 6H6 |
| 2 | CD45 | PerCP | Biolegend | 304026 | HI30 |
| 3 | HLA-DR | PE Fire 640 | Biolegend | 307676 | L243 |
| 4 | CD14 | SPARK Blue 550 | Biolegend | 367148 | 63D3 |
| 5 | CD16 | BUV496 | BD | 612944 | 3G8 |
| 6 | CD3 | BV510 | Biolegend | 344828 | SK7 |
| 7 | TCR- | PerCP-eFluor 710 | Thermo Fisher | 46-9959-42 | B1.1 |
| 8 | CD45RA | BUV395 | BD | 740315 | 5H9 |
| 9 | CD197 CCR7 | BV421 | Biolegend | 353208 | G043H7 |
| 10 | CD56 | BUV737 | BD | 612766 | NCAM 16.2 |
| 11 | CD8 | BUV805 | BD | 612889 | SK1 |
| 12 | CD19 | SPARK NIR 685 | Biolegend | 302270 | HIB19 |
| 13 | CD2 | PerCP-Cy5.5 | Biolegend | 309226 | TS1/8 |
| 14 | CD4 | SPARK YG 581 | Biolegend | 344670 | SK3 |
| 15 | IgD | BV480 | BD | 566138 | IA6-2 |
| 16 | CD11c | Pacific Blue | Biolegend | 301626 | 3,9 |
| 17 | CD127 | APC-R700 | BD | 565185 | HIL-7R-M21 |
| 18 | CD38 | APC Fire 810 | Biolegend | 303550 | HIT2 |
| 19 | CD141 | BB515 | BD | 565084 | 14A |
| 20 | CD39 | BUV661 | BD | 749967 | TU6 |
| 21 | CD27 | APC-Cy7 | Biolegend | 356424 | M-T271 |
| 22 | CD28 | BV650 | Biolegend | 302946 | CD28.2 |
| 23 | IgM | BV570 | Biolegend | 314517 | MHM-88 |
| 24 | IgG | BV605 | BD | 563246 | G18-145 |
| 25 | CD185 (CXCR5) | BV750 | BD | 747111 | RF8B2 |
| 26 | CD183 (CXCR3) | PE-Cy7 | Biolegend | 353720 | G025H7 |
| 27 | CD95 (FAS) | PE-Cy5 | Biolegend | 305610 | DX2 |
| 28 | CD57 | FITC | Biolegend | 359604 | HNK-1 |
| 29 | CD159c (NKG2c) | PE | Biolegend | 375004 | S19005E |
| 30 | CD337 (NKp30) | PE DAZZLE 594 | Biolegend | 325232 | P30-15 |
| 31 | CD279 (PD-1) | BV785 | Biolegend | 329930 | EH12.2H7 |
| 32 | CD196 (CCR6) | BV711 | Biolegend | 353436 | G034E3 |
| 33 | CD24 | PE-Alexa Fluor 610 | Thermo Fisher | MHCD2422 | SN3 |
| 34 | CD25 | PE-Alexa Fluor 700 | Thermo Fisher | MHCD2524 | CD25-3G10 |
| 35 | CD20 | Pacific Orange | Thermo Fisher | MHCD2030 | HI47 |
| 36 | CD66b | Alexa647 | Biolegend | 392911 | 6/40C |
| 37 | CD195 (CCR5) | BUV563 | BD | 741401 | 2D7/CCR5 |
| 38 | CD185 (CXCR5) | BV750 | BD | 747111 | RF8B2 |
| 39 | CD314 (NKG2D) | BUV615 | BD | 751232 | 1D11 |
| 40 | DAPI | DAPI | Thermo Fisher | D1306 |  |
