# Supplementary material for: ER proteostasis failure in HYOU1 deficiency alters B cells, neutrophils, and interferon signalling
Source: J Hum Immun. 2026 Sep 7;2(6):e20250234. doi: 10.70962/jhi.20250234 (PMC13549398; doi:10.70962/jhi.20250234)
Supplement: Supplementary file 6 [file jhi_20250234_tables5.docx]

**Table S5. Sample size for patients and controls per tissue and experiment.**

| Sample type | Experiment | Patient samples | Controls (n) | Control details |
| --- | --- | --- | --- | --- |
| HDF | Bulk RNAseq (unstressed) | Patient 1 (n=1) | n=4 | HDF Control 1 (age-matched), HDF Controls 2, 3, 4 (neonatal)* |
|  | Proteomics  (unstressed) | Patient 1 (n=1) | n=4 | HDF Control 1 (age-matched), HDF Controls 2, 3, 4 (neonatal)* |
|  | RT-qPCR  (unstressed) | Patient 1 (n=1) | n=4 | HDF Control 1 (age-matched), HDF Controls 2, 3, 4 (neonatal)* |
|  | Western blot  (unstressed) | Patient 1 (n=1) | n=4 | HDF Control 1 (age-matched), HDF Controls 2, 3, 4 (neonatal)* |
|  | Bulk RNAseq (tunicamycin stress test) | Patient 1 (n=3 biological replicates) | n=3 (three biological replicates for each control) | HDF Control 1 (age-matched), HDF Controls 2, 3 (neonatal)* |
|  | RT-qPCR  (tunicamycin stress test) | Patient 1 (n=3 biological replicates) | n=3 (three biological replicates for each control) | HDF Control 1 (age-matched), HDF Controls 2, 3 (neonatal)* |
|  | Western blot  (tunicamycin stress test) | Patient 1 (n=3 biological replicates) | n=3 (three biological replicates for each control) | HDF Control 1 (age-matched), HDF Controls 2, 3 (neonatal)* |
| Bone marrow | Bulk RNAseq | Patient 1 (n=1) | n=1 | BM Control 5 |
|  | scRNAseq | Patient 1 (n=1) | n=4 | BM Control 5; three publicly available controls from Oetjen et al., 2018 (GSM3396162_B, GSM3396163_C1, GSM3396185_W) |
| Blood | Bulk RNAseq (PBMC) | Patient 1 (n=3 biological replicates) | n=5 | Blood Controls 3, 4, 6, 7, 8 |
|  | Proteomics (PBMC) | Patient 1 (n=3 biological replicates) | n=8 | Blood Controls 1, 2, 3, 4, 6, 7, 8, 9 |
|  | scRNAseq | Patient 1 (n=3 biological replicates) | n=4 | Blood Controls 2, 3, 8, and patient's healthy brother |
|  | RT-qPCR  (PBMC) | Patient 1 (n=3 biological replicates) | n=3 | Blood Controls 10,11, and 12 |

* HDF control numbering (HDF Controls 1-4) is independent of and distinct from blood/bone marrow control numbering (Blood/BM Controls 1-9), which refer to separate cohorts of healthy individuals. HDF, human dermal fibroblast; PBMC, peripheral blood mononuclear cell; scRNAseq, single-cell RNA sequencing; BM, bone marrow.
